# COVID-19 legislative response and challenges in Republic of Korea

**DOI:** 10.3389/fpubh.2026.1800597

**Published:** 2026-05-13

**Authors:** Hyun-Young Shin, Moran Ki

**Affiliations:** 1 Department of Family Medicine, Seoul St. Mary’s Hospital, College of Medicine, The Catholic University of Korea, Seoul, Republic of Korea; 2 National Cancer Center, Goyang-si, Republic of Korea

**Keywords:** COVID-19, governance, legislation, pandemic, preparedness, public health policy

## Abstract

This narrative review systematically analyzes the longitudinal evolution of South Korea’s legal and institutional frameworks from the 2015 MERS outbreak through the COVID-19 pandemic. The study introduces the “Pandemic Response Pentad,” an original conceptual model positioning Legislation as the foundational core, mediated by Governance, to drive three interconnected field operations. Based on this framework, Korea’s infectious disease control system is evaluated across four operational domains: (1) governance reform and personnel structure enhancement, (2) epidemiological response capabilities and data utilization, (3) medical response system, including vaccination programs and supply stockpiling, and (4) social response mechanisms, encompassing social distancing and the protection of vulnerable populations. While rapid legislative enactments enabled effective disease control, they also generated profound ethical tensions regarding fundamental human rights. To mitigate these unintended socioeconomic consequences, Korea institutionalized extensive statutory compensation mechanisms for healthcare facilities, small business owners, and vaccine injuries. The findings highlight that advancing national research infrastructure and establishing policy-oriented think tanks for future pandemic preparedness strictly require proactive legislative backing. Ultimately, this study provides valuable insights into the critical interplay between legal mandates and institutional resilience in global health crisis management.

## Introduction

The Republic of Korea (hereafter Korea)'s response to COVID-19 has garnered significant interest from the international community. Although Korea attained a prominent position in Bloomberg’s COVID-19 Resilience Ranking in 2022, this achievement can be attributed to a combination of factors, including active participation by citizens, government actions rooted in the 3 T (Test-Trace-Treat) strategy, and consistent public communication efforts (1, 2). Additionally, the legislative branch played a crucial role by establishing institutional mechanisms and legal frameworks, enabling a flexible and coordinated response to the evolving circumstances (3).

Korea previously experienced a substantial Middle East Respiratory Syndrome (MERS) outbreak (172 confirmed cases, 27 deaths in 2015) outside the Middle East, which served as a catalyst for early restructuring of its infectious disease management systems (4, 5). While MERS primarily exhibited hospital-based transmission (5), COVID-19 (2020–2023: 34,572,554 confirmed cases, 30,605 deaths) posed unique challenges due to its pattern of community-based transmission, necessitating further adaptation of institutional and legal approaches (1, 6). Addressing COVID-19 in Korea required coordinated efforts from citizens, healthcare providers, government entities, and legislative initiatives to build a strengthened infectious disease response system. This collaborative model was instrumental in managing both health and socioeconomic impacts of the pandemic. Furthermore, the response phase underscored critical issues requiring further attention, such as social challenges, the protection of human rights, safeguarding personal data, and health security concerns, including vaccine sovereignty (the ability to independently develop and manufacture vaccines).

The establishment of national systems and frameworks for public engagement derived from experiences with infectious diseases can also be seen in other contexts, for example, in the United States’ management of the Ebola virus (7). Insights from the Ebola response highlight that several components of public health preparedness—including governance structures for infectious disease management, public communication protocols, quarantine implementation, surveillance systems, and environmental controls—are integral to enhancing infectious disease response capacity (7). Current evidence demonstrates that future events akin to COVID-19 remain possible, with their emergence likely affected by factors such as climate dynamics and greater global interconnectivity enabled by contemporary transportation and communication networks.

Accordingly, it is important to critically assess present legislative frameworks for pandemic preparedness, identify areas requiring refinement, and anticipate challenges that may arise (8). This research aims to examine laws enacted during Korea’s MERS outbreak and the COVID-19 pandemic by thematic category and propose recommendations for strengthening infectious disease response mechanisms for future public health emergencies.

## Materials and methods

### Data collection and selection criteria

This research examined legislative modifications in Korea using official resources, primarily the National Law Information System (9), and the National Assembly Bill Information System (10). The study period spanned from the post-MERS era in 2015 to the post-COVID-19 era in 2025. The inclusion criteria encompassed all legal provisions revised during this period that directly governed infectious disease management (e.g., Infectious Disease Control and Prevention Act, Government Organization Act, Special Act on Promotion of the Development and Emergency Supply of Medical Products in Response to Public Health Crisis, and Special Act on Compensation for Damage Related to COVID-19 Vaccination.) Provisions not directly related to infectious disease crises were excluded. The progression of legislative changes was corroborated by consulting the 2015 MERS White Paper (4), and COVID-19 White Paper (11), both published by the Korea Disease Control and Prevention Agency (KDCA).

### Cross-verification and categorization process

To ensure analytical rigor and minimize selection bias in this narrative review, text synthesis and categorization (coding) were conducted by a panel of three experts. These individuals played crucial roles in both government and parliamentary legislative processes during the MERS and COVID-19 outbreaks. The experts first independently assessed the chronology and substance of the legislative revisions. Subsequently, they utilized a rigorous cross-check approach to resolve any discrepancies and reach a consensus. Through this consensus, they systematically identified relevant subject categories and arranged the legal articles accordingly. To ensure complete transparency and reproducibility, the specific types of relevant legislation and their corresponding legal articles, meticulously categorized by each response domain, are provided in the Supplementary Tables S1–S8.

### Analytical framework: the pandemic response pentad

The analytical framework of this study, conceptualized as the “Pandemic Response Pentad,” is an original concentric model developed *de novo* by the authors based on empirical consensus from managing the MERS and COVID-19 outbreaks.

The theoretical justification for this model is rooted in the varying scales of public health crises. While localized outbreaks may be managed solely through epidemiological and medical interventions, a large-scale, prolonged pandemic inevitably necessitates a comprehensive social response, such as social distancing, business restrictions, and loss compensation. Because such extensive interventions inherently restrict fundamental human rights and economic freedoms, they cannot be implemented without robust statutory backing.

Therefore, the pentad consists of five interconnected elements with Legislation positioned at the center, serving as the foundational core to safeguard public health through legal enforcement and compensation.

Operating directly upon this core is Governance, which ensures a unified response via leadership, coordination, and resource management. This mediating layer drives three interconnected field operations: the Epidemiological response (focused on incidence control), the Medical response (prioritizing mortality control), and the Social response (concentrating on impact mitigation).

**Figure 1:**
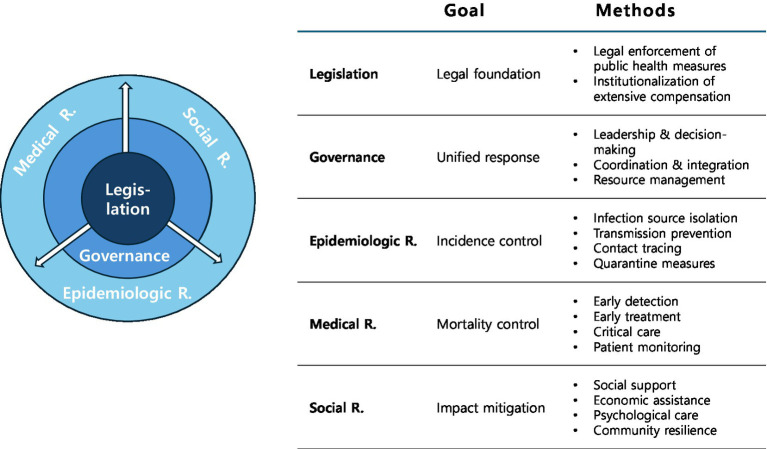
Pandemic response pentad: a concentric model intergrating legal foundation and field operations.

As illustrated by the directional arrows, legislative influence flows outward through governance to the field responses. Crucially, these arrows intentionally do not sever the outer ring, emphasizing that the three field responses are not fragmented entities. Rather, they are highly interconnected domains that continuously communicate and function organically as an integrated system during a public health crisis (Figure 1).

## Results

### COVID-19 timeline and key events

**Figure 2:**
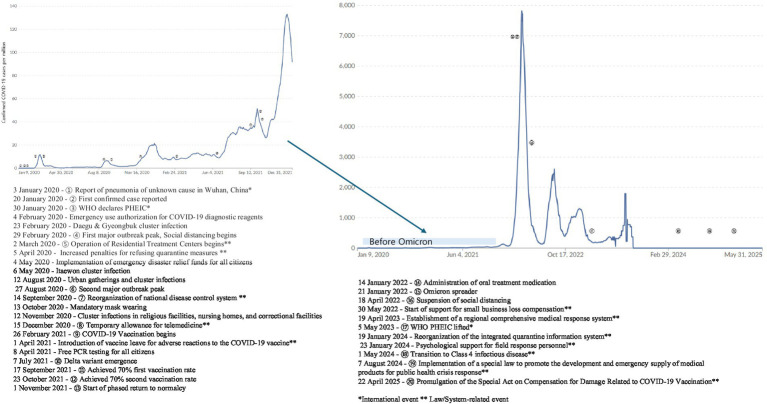
Confirmed COVID-19 cases per million people with major events in the Republic of Korea (2020–2025).

Following the initial COVID-19 case reported in Wuhan on January 3, 2020, Korea identified its first case on January 20. After WHO declared a PHEIC on January 30, Korea implemented social distancing measures in February during the initial outbreak peak. Community Treatment Centers were established in March 2020, and in response to a second wave in August, the KDCA’s authority was enhanced, dual vice-ministers were introduced in MOHW, the National Institute of Infectious Diseases was formed, and financial assistance for living expenses began in September. Temporary approval for telemedicine services was granted in December 2020. Vaccination commenced in February 2021, resulting in 70% first-dose coverage by September and achievement of second-dose targets by October, allowing a phased return to normal activities in November. Despite an extensive Omicron wave in January 2022, disease severity markedly declined. Social distancing measures were discontinued in April 2022, the WHO rescinded the PHEIC in May 2023, and Korea classified COVID-19 as a Class 4 disease in May 2024. Subsequently, the Special Act for Emergency Medical Products Development and Supply was enacted in August 2024, and the Special Act on COVID-19 Vaccination Damage Compensation was introduced in April 2025 (Figure 2).

### Governance reform and personnel structure enhancement

Korea’s governance structure for infectious disease response experienced substantial reform in the wake of the MERS and COVID-19 crises. During the MERS event, new legislation permitted the deployment of epidemiological investigation officers—roles analogous to the US CDC’s Epidemic Intelligence Service [EIS] officers—within the Ministry of Health and Welfare (MOHW) and 16 local governments, boosting capabilities for virus tracking and community transmission assessment. The infectious disease classification system was restructured to categorize novel infectious diseases as Class 1, and the expertise within the Infectious Disease Control Committee was reinforced by increasing membership from 20 to 30.

Following COVID-19, significant organizational reforms were enacted via amendments to the Government Organization Act. These reforms included: (1) granting the KDCA independent agency status, (2) designating a dedicated Second Vice Minister for healthcare within the MOHW, (3) establishing five Regional Centers for Disease Control and Prevention, and (4) founding the National Institute of Infectious Diseases (NIID). The number of epidemiological investigation officers expanded from 30 to 100, with capacity for deployment across 250 municipal districts (Table 1; Supplementary Table S1).

**Table 1:** Legislative amendments in governance and epidemiological response system^a^.

Classification	Post-MERS (2015–2020)	COVID19-Era (2020–2025)
Governance reform and personnel structure	Legal basis for placement of epidemiological intelligence officers (EIS): Ministry of Health and Welfare (30 officers), local governments (2 officers) Reorganization of infectious disease classification system (emerging infectious diseases classified as Class 1) Legal grounds for establishment and operation of emergency operations center Enhancement of Infectious Disease Control Committee (KCDC Director as chairperson, committee size increased from 20 to 30 members)	Government Organization Act: Establishment of the KDCA as an independent agency status, RCDC, and the position of the second vice minister of MOHW Legal basis for placement of disease control officers and EIS officers at local level; expansion of MOHW EIS officers (30 to 100) Temporary deployment authority for disease control officers granted to provincial and local governors Specification of provisional EIS officers’ scope, authority, and duties Mandatory infectious disease training for national and local public servants Addition of Expert Committee on Infectious Disease Laboratory Diagnostics Legal grounds for integrated infectious disease management information system
Epidemiological response	Legal basis for prompt public disclosure of patient information during disease spread (movement history, transportation, medical facilities visited, contact tracing data) Legal basis for paid leave during hospitalization or quarantine Legal authority for public officials or judicial police to escort non-compliant individuals for mandatory screening and isolation Legal framework for designation of quarantine facilities for close contacts	Mandatory testing requirements and penalties for Class 1 infectious disease suspects (free testing) Legal basis for isolation, investigation, examination, and hospitalization of Class 1 disease suspects; enhanced penalties for isolation non-compliance Extension of authority to local governments for patient location data requests (expanded from central to local) Healthcare facility access to immigration records via NHIS system; mandatory verification during prescription/dispensing Detailed specifications for information disclosure during disease crisis (including appeal procedures) Extended disclosure obligations to local authorities; removal of unnecessary information upon achieving disclosure objectives Annual public reporting requirement for personal information processing

KDCA, Korea Disease Control and Prevention Agency; RCDC, Regional Centers for Disease Control and Prevention; MERS, Middle East Respiratory Syndrome; MOHW, the Ministry of Health and Welfare; NHIS, the National Health Insurance Service.

### Epidemiological response

Evaluation of Korea’s epidemiological response framework revealed systematic advancements driven by two major infectious disease outbreaks. Over time, the framework progressed from basic transparency protocols to a fully developed, technology-integrated approach.

#### Post-MERS legislative framework

Reforms following MERS introduced essential control strategies by amending the Infectious Disease Control and Prevention Act. Central changes involved: (1) mandatory public disclosure of patient movement routes, transportation, and contact tracing details, (2) established procedures for isolation facility designation, (3) legal authority to enforce diagnostic testing, and (4) provision of paid leave for those under isolation to encourage compliance (Table 1).

#### COVID-19 era enhancement

The COVID-19 pandemic led to extensive updates of the existing framework. Notable measures included: (1) offering free diagnostic testing for Class 1 infectious diseases with compulsory compliance, (2) reinforcing isolation protocols with stricter penalties, (3) empowering provincial governors and local authorities with decentralized investigation rights, including access to location data, and (4) establishing precise standards and steps for information disclosure.

The revised framework incorporated privacy safeguards by (1) restricting disclosure to essential information, (2) establishing mandatory data deletion procedures upon fulfillment of public health objectives, and (3) instituting annual public reporting on personal data handling. Integration with the healthcare system was supported by leveraging the NHIS information network and travel history records for evidence-based clinical management.

These extensive framework revisions transformed Korea’s epidemiological response into a unified system that effectively balances public health needs with the imperative of privacy protection (Table 1; Supplementary Table S2).

### Medical response

#### Medical response system enhancement

The post-MERS policy introduced a compensation mechanism for medical facilities caring for infectious disease cases, with the Loss Compensation Review Committee responsible for compensation decisions. This policy incentivized robust engagement of medical institutions in infectious disease management.

COVID-19 era system adaptations comprised: (1) the designation of Community Treatment Centers in addition to hospital facilities, (2) the introduction of an advance partial payment system for compensating losses, (3) provision of financial support to healthcare workers, and (4) the establishment of specialized infectious disease hospitals across five major regions.

During the Omicron surge, significant changes included: (1) the introduction of a legal framework supporting home isolation recommendations, (2) temporary authorization of telemedicine services, (3) implementation of psychological support programs for frontline workers and patients, and (4) development of patient transfer protocols that considered associated costs. Management of foreign patient treatment followed reciprocity principles (Table 2; Supplementary Table S3).

**Table 2:** Legislative amendments in medical response and vaccination systems^a^.

Classification	Post-MERS (2015–2020)	COVID19-Era (2020–2025)
Medical response system enhancement	Establishment and designation of specialized infectious disease hospitals and research hospitals Compensation provision for medical institutions treating infectious disease patients: establishment of loss compensation committee for deliberation and resolution	Legal basis for patient transport; self-pay requirement for transport refusal Authority for facility requisition during outbreaks (hospitals, training centers, accommodations for Residential Treatment Centers) Cost-bearing provisions for foreign patients (reciprocity principle) Legal framework for temporary telemedicine services Regional establishment/designation of specialized infectious disease hospitals Financial support framework for healthcare workers Psychological support and related expense coverage during disease crisis Provision for partial advance payment of loss compensation (note: free treatment and living expense support for isolated individuals)
Vaccination program development	Regulations on establishment and operation of Integrated Vaccination Information Management System	Paid leave provision for worker vaccination; government cost support Special Act on Vaccination Injury Compensation Note: Free vaccination and adverse reaction compensation
Medical supply management, stockpiling, and procurement		Inclusion of stockpiling and management of pharmaceuticals/equipment in National Infectious Disease Master Plan Provincial/municipal authorities’ responsibility for stockpiling and managing pharmaceuticals/equipment Export ban and penalties for pharmaceuticals/medical supplies during Class 1 infectious disease outbreaks Legal basis for advance purchase of developmental vaccines/pharmaceuticals; protection of officials from disadvantages MFDS fast-track approval system for products during public health emergencies

MERS, Middle East Respiratory Syndrome; MFDS, Ministry of Food and Drug Safety.

#### Vaccination program development and adverse event compensation systems

Post-MERS reforms aimed to develop an integrated vaccination information management system to improve the efficiency of immunization data handling.

COVID-19 era measures comprised: (1) mandatory paid leave for vaccination with government financial assistance, and (2) the adoption of the Special Act on Vaccination Damage Compensation (2025), ensuring comprehensive support for adverse events to vaccination (Table 2; Supplementary Table S4).

#### Medical supply management, stockpiling, and procurement

COVID-19 highlighted the need for comprehensive supply management reforms including: (1) strengthened emergency medical supply regulations in response to mask shortages, (2) integration of stockpiling policies into the Basic Infectious Disease Response Plan, (3) approval for local government-led stockpile management, and (4) the imposition of export restrictions during Class 1 disease crises.

Optimization measures were implemented including: (1) the launch of the Ministry of Food and Drug Safety (MFDS) fast-track review process, (2) the establishment of a legal basis for advance purchase agreements, and (3) introduction of protections for procurement officials to support prompt actions (Table 2; Supplementary Table S5).

### Social response

#### Social distancing and compliance measures

Post-MERS approaches created institutional structures for enforcing preventive actions, such as penalties for not wearing masks, to strengthen community adherence to public health guidance.

COVID-19 era initiatives built on this structure by: (1) applying social distancing principles, (2) implementing vaccine pass requirements for participation in certain activities, (3) enacting legal grounds for suspending operational licenses of facilities, and (4) enabling liability claims for infections resulting from non-compliance. Compensation mechanisms were put in place for small business operators and self-employed individuals economically affected by social distancing or quarantine (Table 3; Supplementary Table S6).

**Table 3:** Legislative amendments in social response and vulnerable population protection^a^.

Classification	Post-MERS (2015–2020)	COVID19-Era (2020–2025)
Social distancing	Legal basis for mandating preventive measures (e.g., mask wearing, social distancing, vaccine pass) with administrative fines for violations Authority to order business suspension for facilities/establishments violating disease prevention protocols Liability provisions for damage claims against those spreading infectious diseases through violation of prevention measures	Compensation for losses to self-employed/small business owners & individuals under quarantine
Vulnerable population protection and human rights	Legal basis for living expenses and financial support for persons under hospitalization or quarantine	Provision of masks and other measures for vulnerable populations during alert levels of ‘Caution’ or higher Framework for response strategies by types of vulnerable populations and social welfare facilities Expanded scope of vulnerable populations: low-income groups, children/elderly/disabled persons using social welfare facilities Regulations for exceptional outings (voting rights, exams, funerals, etc.) of infectious disease patients with notification of precautionary measures
Infectious disease R&D		Legal basis for KDCA Commissioner to analyze collected data and utilize it for infectious disease research Establishment of National Institute of Infectious Diseases under KDCA with authority to allocate government contributions

KDCA, Korea Disease Control and Prevention Agency; MERS, Middle East Respiratory Syndrome.

#### Protection of vulnerable populations

Post-MERS amendments introduced both financial and living assistance systems for those hospitalized or quarantined, aimed at supporting compliance with treatment and isolation requirements.

COVID-19 response measures improved protection by: (1) supplying preventive resources to vulnerable groups, (2) developing targeted strategies for social welfare institutions, (3) broadening the definition of vulnerable populations to encompass low-income groups, children, older adults, and persons with disabilities, and (4) implementing a basic framework for the protection of social rights that permitted certain outings under strict precautionary protocols (Table 3; Supplementary Table S7).

#### Infectious disease research and development

Early post-MERS regulations concentrated on the designation and operation of specialized hospitals for infectious disease research and care.

During the COVID-19 era, research capabilities were further advanced by: (1) granting KDCA the authority to conduct infection-related research utilizing collected data, (2) founding the NIID within KDCA with the ability to allocate research funding, and (3) developing a framework to facilitate government-led and public-private research collaborations (Table 3; Supplementary Table S8).

## Discussion

This study revealed that Korea’s legislative response to COVID-19 targeted four primary domains: (1) reinforcing governance and oversight across central and local governments, (2) improving epidemiological investigative capacity, (3) strengthening clinical response systems, vaccination strategies, and essential stockpiles, and (4) elevating social measures by enhancing social distancing protocols, supporting vulnerable groups, protecting human rights, and increasing infectious disease research capabilities.

Institutional reforms following the MERS outbreak mainly concentrated on countering hospital-based transmissions, advancing epidemiological investigations, boosting clinical response capacity, improving hospital infection control, enhancing emergency room protocols, and instituting compensation frameworks for hospital losses. In contrast, the COVID-19 response addressed wider community transmission, strengthening and expanding epidemiological responses, such as the management of infected persons and contacts, and implementing community-wide social distancing. The scale and duration of the pandemic necessitated more robust social policies, including harmonized governance mechanisms, human rights safeguards, increased research and development, and broader stockpile strategies.

Korea’s effective initial COVID-19 response was grounded in post-MERS legal frameworks, which facilitated transparent information sharing, effective public communication, and swift initiation of testing. Nonetheless, as the pandemic persisted and new issues arose, notably in safeguarding vulnerable populations, supporting small business operators and self-employed individuals, and managing adverse vaccination effects, the legislative focus was extended to address these additional challenges.

The COVID-19 pandemic, as both a natural and social disaster, prompted a notable response from the National Assembly. Members from both ruling and opposition parties promptly addressed urgent needs through legislative consensus, allowing the executive branch to enact timely measures and safeguard citizens with targeted interventions.

### Governance reform and personnel structure enhancement

As the second country after China to face a COVID-19 outbreak in 2020, Korea reorganized its foundational response system to facilitate a more robust governmental role in infectious disease management. Measures included upgrading the Korea Centers for Disease Control and Prevention (KCDC) to the KDCA, enhancing its status as an independent agency, establishing a designated vice minister for healthcare, and expanding administrative powers for both central and local authorities.

The COVID-19 pandemic highlighted differences in healthcare system preparedness among countries, prompting a global movement toward reinforcing government oversight in healthcare (12). Comparative studies with Japan indicated that administrative guidance by itself is insufficient, with effective disease management relying on a combination of authoritative directives and enforceable penalties (13). In a similar vein, Canada revised its public health laws to increase government accountability via expanded emergency authority, underscoring the importance of strengthening mechanisms that enhance public confidence in decision-makers (14).

The Ukrainian government enhanced its capacity by swiftly making expert decisions concerning infection transmission, conducting epidemiological monitoring, and securing access to diagnostics and vaccines (15). Malaysia’s response was coordinated through a centralized committee spanning multiple ministries, focusing on five key domains: whole-of-government strategy, cordon sanitaire/lockdown, equitable access, quarantine management, and enforcement (16).

### Epidemiological response; personal information protection and data collection and utilization

The institutional resolution of conflicts between infectious disease surveillance data collection and personal information protection remains a significant national challenge in public health informatics. In Korea, the insufficient release of epidemiological data during the MERS outbreak highlighted the need for improved epidemic response, leading to the introduction of enhanced surveillance practices during COVID-19, such as public disclosure of the movement histories of confirmed cases. Nonetheless, the regulatory framework for collecting and using individual health data in infectious disease surveillance still requires substantial refinement.

The US has implemented more advanced regulatory mechanisms in this area, adopting a comprehensive informed consent framework based on the opt-out model as the core principle for obtaining authorization from data subjects. To promote practical implementation and manage data identifiability, the US system includes dynamic consent protocols. Importantly, the US government sustains health data infrastructures through public-private collaborations, with the National Institutes of Health (NIH) primarily coordinating data sharing for research purposes. This integrated strategy provides important guidance on developing methodological frameworks for health data usage in public health crises (17).

### Medical response

#### Medical response system enhancement

The COVID-19 pandemic prompted substantial regulatory modifications within Korea’s healthcare system. The revision of the Infectious Disease Control and Prevention Act provided legal authorization for temporary telemedicine services during periods of infectious disease alerts, permitting remote consultations and prescription issuance. This regulatory adaptation ensured the ongoing provision of healthcare while reducing infection transmission risks. Furthermore, the Ministry of Food and Drug Safety (MFDS) introduced accelerated review pathways for medical products in public health emergencies, streamlining procedures for both imported and domestic products to expedite market availability.

Comparative international analysis highlights varied regulatory strategies among different countries. In the US, the VALID (Verifying Accurate Leading-edge IVCT Development) Act addressed diagnostic test regulation issues by centralizing management of laboratory-developed and commercial *in vitro* diagnostic tests, thus increasing regulatory adaptability (18). Kenya adopted legislative reforms that prioritized enhancements in the healthcare system’s resilience, including improved financial frameworks, implementation of price controls, and optimization of supply chain management (19).

#### Vaccination program and adverse event compensation systems

Korea’s COVID-19 vaccination rollout was underpinned by regulatory structures promoting accelerated research, development, and authorization processes (20). The development of robust adverse event compensation mechanisms played an important role in reinforcing public trust and engagement in vaccination campaigns (21).

Although Korea’s Infectious Disease Control and Prevention Act has included compensation measures for adverse events related to essential vaccinations since 1995, significant social debates and controversies emerged in response to COVID-19 vaccine adverse events, especially regarding the demonstration of causality as well as the coverage and adequacy of the compensation system (20). Ultimately, with the passage of the Special Act on COVID-19 Vaccination Damage Compensation by the National Assembly in 2025, the compensation system was significantly broadened.

A comparative review of COVID-19 vaccine injury compensation frameworks across 14 countries identified marked differences in approval rates. Japan reported the highest approval rate at 74·29%, whereas Korea and France had moderate approval rates of 26·09% and 27·42%, respectively. The US and UK displayed notably lower approval rates at 3% and 2·64%, respectively (22). Various countries established unique features within their compensation systems, with Germany, Japan, and Taiwan adopting revised criteria for causality assessment. Thailand introduced obligatory primary compensation, while France and Japan designed functional evaluation procedures. Additionally, the UK and France adopted unified assessment standards, and countries such as France, the UK, and Australia created specialized institutional bodies to oversee compensation claims (23).

### Social response

#### Protection of vulnerable populations and human rights

The COVID-19 pandemic underscored challenging tensions between the expansion of governmental authority and the safeguarding of the rights of individuals and minority groups in diverse legal and cultural settings. This interplay differed depending on each country’s societal, legal, and cultural circumstances, necessitating a thorough analysis of how to balance urgent public health demands with the protection of fundamental rights.

The Korean context posed distinct challenges and prompted adaptive responses. Korea’s disaster management system, originally centered on natural disasters, was substantially revised after the MERS outbreak to address infectious disease threats. However, it is imperative to critically reflect on the ethical tensions and unintended consequences that emerged during this legislative evolution. While the rapid enactment of robust legal frameworks enabled effective disease control, it simultaneously generated profound societal friction. The extensive implementation of social distancing mandates, business restrictions, and strict quarantine measures inevitably clashed with fundamental human rights, including the freedom of movement and economic liberty.

Although COVID-19 impacted the entire population, these state-mandated interventions disproportionally exposed the vulnerabilities of certain groups, such as those who are economically disadvantaged, small business owners, young children, older adults, persons with disabilities, homeless individuals, and group facility residents (24). As a result, the Korean government enacted focused interventions, broadening the criteria for infection-vulnerable groups and introducing specialized support strategies, such as strengthened mask distribution frameworks and facility-specific infection prevention protocols.

Global experiences offered important perspectives on safeguarding human rights during public health crises. In Ireland, emergency laws that extended authorities for detention, isolation, and the arrest of suspected infection carriers were subjected to constitutional scrutiny, though the High Court ultimately upheld them (25). The German response illuminated ethical dilemmas in the allocation of medical resources, especially in the context of disability rights. Their strategy engaged both survival probability criteria and principles of non-discrimination, utilizing a mix of lottery approaches and first-come-first-served procedures, with continuous attention to the protection of disability rights (26).

North American jurisdictions faced specific challenges concerning racial equity in the enforcement of public health measures. Documented discriminatory practices targeting Black communities during COVID-19 containment increased the prioritization of social justice and accountability in public health strategies. This prompted policy reform at upstream levels, organizational changes, and updated approaches to resource distribution in an effort to correct systemic inequities (27). South Africa’s experience highlighted the necessity for legislative adaptability when strict lockdowns unintentionally heightened risks of abuse and neglect among children. The government responded by adopting targeted social protection for high-risk children while preserving critical legal supports (28).

These varied international responses highlight a key conclusion: the extension of infectious disease control measures exposed gaps in human rights protections for vulnerable and minority populations, necessitating more agile legislative frameworks to strengthen social resilience and address national contexts. Consequently, the pandemic prompted renewed attention to the interplay between emergency authority and human rights guarantees, with a focus on safeguarding the most at-risk groups.

#### Economic loss compensation framework

To address these deep ethical tensions and mitigate the unintended socio-economic damages caused by prolonged public health interventions, Korea established a unique statutory system for comprehensive loss compensation. This framework was not merely an administrative adjustment, but a necessary legal remedy providing support to medical institutions, individuals in quarantine, and, notably, self-employed persons and small business operators whose livelihoods were directly curtailed by government mandates. This statutory model was distinct from the informal support mechanisms adopted in other countries. Analysis of international economic compensation strategies demonstrated significant variation. Japan relied primarily on its existing H1N1 pandemic laws with minor updates, while Germany prioritized maintaining employment and offered direct business support. Meanwhile, France responded by continuously adapting its COVID-19 Solidarity Fund compensation through monthly regulatory decrees (29).

### Study limitations and strengths

This study has several limitations that must be recognized. First, by adopting a narrative review approach that largely concentrated on healthcare-related statutes and societal response measures specific to COVID-19—rather than conducting a comprehensive systematic review of all legislative domains—the possibility of methodological selection bias cannot be excluded. This selective focus implies that certain peripheral legal adaptations might have been overlooked, which could potentially affect the overall generalizability and robustness of the synthesized evidence. Second, although the analysis provided a comprehensive evaluation of primary legislation, it considered only Enforcement Decrees and Enforcement Rules. Third, this research focused solely on enacted laws, omitting proposed bills that either lacked sufficient review or were not enacted; however, the numerous recommendations for institutional improvement made during the pandemic era deserve further analysis for their potential relevance. Fourth, this study relies on a qualitative, structural analysis of legislative frameworks rather than quantitatively evaluating their direct effectiveness or epidemiological outcomes. Because the implementation and impact of legal provisions are deeply rooted in Korea’s specific socio-political and healthcare context, quantifying these institutional inputs remains methodologically challenging. Therefore, direct application of these legislative cases to other countries requires careful consideration and flexible adaptation to unique national circumstances.

Despite these limitations, the study identifies several notable strengths that significantly enhance the field. Firstly, it offers a thorough and systematic overview of Korea’s COVID-19 legislative framework, which serves as an important reference for international collaboration and for preparing the legislative prerequisites for future pandemic responses. Secondly, the study analyzes the institutional progression following two major infectious disease outbreaks (MERS and COVID-19), demonstrating the evolution and increased capability of Korea’s public health response infrastructure. Thirdly, it provides an extensive review of the governance structure incorporating three essential response areas—epidemiologic surveillance, medical countermeasures, and social interventions—which may serve as a reference model for cross-national pandemic strategies.

## Conclusion

This study highlights the critical interplay between legislative foundations and institutional developments within Korea’s COVID-19 response system. Given that the management of infectious disease crises necessitates expansive preparedness, current circumstances demand both critical retrospective assessment and proactive future planning.

Our findings indicate that while advancing national research infrastructure, implementing structured training programs for infectious disease professionals, and reinforcing global surveillance systems are key institutional priorities, these initiatives cannot be realized without robust and proactive legislative preparation. Similarly, the establishment of policy-oriented think tanks to augment conventional research bodies requires explicit statutory backing to effectively create comprehensive response frameworks and assess societal consequences during public health crises.

Therefore, to establish a more advanced response system for the next pandemic, policymakers must prioritize these legislative tasks well in advance, ensuring that every institutional mechanism is firmly grounded in law. Lessons drawn from Korea’s adaptation to MERS and COVID-19 furnish valuable guidance for international pandemic preparedness. The legal frameworks and subsequent institutional responses formed during these outbreaks provide significant precedents for improving public health emergency preparedness, emphasizing the necessity for ongoing legislative innovation to address future public health threats.
