# Supplementary material for: COVID-19 legislative response and challenges in Republic of Korea
Source: Front Public Health. 2026 May 13;14:1800597. doi: 10.3389/fpubh.2026.1800597 (PMC13212051; doi:10.3389/fpubh.2026.1800597)
Supplement: Supplementary file 1 [file Data_Sheet_1.DOCX]

| **Supplementary Tables**   1. Table S1. Governance Reform and Personnel Structure | | |
| --- | --- | --- |
| TITLE | ARTICLE | CONTENT |
| GOVERNMENT ORGANIZATION ACT | Article 39② (Ministry of Health and Welfare) | (2) In order to administer duties concerning infectious diseases such as prevention and quarantine and duties concerning investigation, test, and research of various diseases, the Korea Disease Control and Prevention Agency shall be established under the Minister of Health and Welfare. |
| ORGANIZATION CHART OF THE MINISTRY OF HEALTH AND WELFARE AND ITS AFFILIATED AGENCIES (ENFORCEMENT RULES) | Article 4-2 (Operation of Multiple Vice Ministers) | ① The Ministry of Health and Welfare shall have a First Vice Minister and a Second Vice Minister, and in the event that the Minister is unable to perform their duties due to unavoidable circumstances, the First Vice Minister and then the Second Vice Minister shall act in their place.  ③ The Second Vice Minister shall assist the Minister in matters related to the Health and Medical Policy Office, the Health Insurance Policy Bureau, the Health Policy Bureau, and the Health Industry Policy Bureau. [This Article was established on September 11, 2020.]" |
| INFECTIOUS DISEASE CONTROL AND PREVENTION ACT | Article 7 (Formulation of Plans for Prevention and Control of Infectious Diseases) | (1) The Commissioner of the Korea Disease Control and Prevention Agency shall formulate and implement in consultation with the Minister of Health and Welfare a master plan for preventing and controlling infectious diseases (hereinafter referred to as "master plan") for every five years.  <Amended on Jan. 18, 2010; Aug. 11, 2020>  (3) The Special Metropolitan City Mayor, a Metropolitan City Mayor, a Special Self-Governing City Mayor, a Do Governor, a Special Self-Governing Province Governor (hereinafter referred to as "Mayor/Do Governor"), and the head of a Si/Gun/Gu (referring to the head of an autonomous Gu; hereinafter the same shall apply) shall formulate and implement an implementation plan, based on a master plan. <Amended on Jun. 13, 2023>  (4) The Commissioner of the Korea Disease Control and Prevention Agency, a Mayor/Do Governor, or the head of a Si/Gun/Gu may request relevant administrative agencies or associations to provide data necessary for formulating and implementing master plans or implementation plans under paragraph (3). <Amended on Jan. 18, 2010; Aug. 11, 2020> |
|  | Article 9 (The Infectious Disease Control Committee) | (1) An Infectious Disease Control Committee (hereinafter referred to as the "Committee") shall be established under the Korea Disease Control and Prevention Agency to deliberate on major policies on the prevention and control of infectious diseases. <Amended on Jan. 18, 2010; Aug. 11, 2020>  (2) The Committee shall deliberate on the following: <Amended on Mar. 18, 2014; Dec. 2, 2016; Dec. 3, 2019; Dec. 15, 2020; Mar. 9, 2021; Jun. 10, 2022> |
|  | Article 40-5 (Integrated Infectious Disease Control Information System) | (3) An infectious disease information system may be utilized by electronically linking it to the following information systems; in such cases, data or information that may be collected through such linkage shall be limited to those for the prevention, management and treatment of patients of an infectious disease, etc.:   <Amended on Jan. 17, 2023; Aug. 16, 2023> |

Table S2. Enhancement of Epidemiological Response Capacity,

Title: INFECTIOUS DISEASE CONTROL AND PREVENTION ACT

| ARTICLE | CONTENT |
| --- | --- |
| Article 2 (Definitions) | 5. The term "Class 4 infectious disease" means any of the following infectious diseases that require sentinel surveillance to investigate whether they are epidemic, other than Classes 1 through 3 infectious diseases: Provided, That infectious diseases designated by the Commissioner of the Korea Disease Control and Prevention Agency shall be included: |
| Article 2 (Definitions) | 15-2. The term "person suspected of contracting an infectious disease" means any of the following: (a) A person (hereinafter referred to as "contact") who comes into contact with or is suspected of coming into contact with a patient or probable patient of an infectious disease or pathogen carrier (hereinafter referred to as "patient of an infectious disease, etc."); (b) A person who has stayed in, or passed through, a quarantine inspection required area or strict quarantine inspection required area defined in subparagraph 7 or 8 of Article 2 of the Quarantine Act, and may have contracted an infectious disease; (c) A person who has been exposed to risk factors, such as infectious pathogens, and may have contracted an infectious disease; |
| Article 4 (Responsibilities of the State and Local Governments) | (2) The State and local governments shall perform the following projects for preventing and controlling infectious diseases: <Amended on Mar. 18, 2014; Jul. 6, 2015; Mar. 4, 2020; Dec. 15, 2020>  (4) The State and local governments shall share the relevant information with medical institutions and medical personnel's associations prescribed in the Medical Service Act in order to monitor and prevent the outbreak of infectious diseases. <Newly Inserted on Jul. 6, 2015> |
| Article 7 (Formulation of Plans for Prevention and Control of Infectious Diseases) | (1) The Commissioner of the Korea Disease Control and Prevention Agency shall formulate and implement in consultation with the Minister of Health and Welfare a master plan for preventing and controlling infectious diseases (hereinafter referred to as "master plan") for every five years.  <Amended on Jan. 18, 2010; Aug. 11, 2020>  (3) The Special Metropolitan City Mayor, a Metropolitan City Mayor, a Special Self-Governing City Mayor, a Do Governor, a Special Self-Governing Province Governor (hereinafter referred to as "Mayor/Do Governor"), and the head of a Si/Gun/Gu (referring to the head of an autonomous Gu; hereinafter the same shall apply) shall formulate and implement an implementation plan, based on a master plan. <Amended on Jun. 13, 2023>  (4) The Commissioner of the Korea Disease Control and Prevention Agency, a Mayor/Do Governor, or the head of a Si/Gun/Gu may request relevant administrative agencies or associations to provide data necessary for formulating and implementing master plans or implementation plans under paragraph (3). <Amended on Jan. 18, 2010; Aug. 11, 2020> |
| Article 8 (Operation of Organizations Supporting Infectious Disease Control Projects) | (1) The Commissioner of the Korea Disease Control and Prevention Agency and a Mayor/Do Governor may establish an organization supporting infectious disease control projects which consists of private professionals, etc. in order to support the implementation of mater plans and implementation plans under Article 7 and international cooperation affairs, etc. <Amended on Jan. 18, 2010; Aug. 11, 2020; May 19, 2023> |
| Article 11 (Reporting by Physicians) | 4. Where a person suspected as a patient of an infectious disease refuses infectious pathogen testing.  (2) Where a staff member of an institution for confirming infectious pathogens referred to in Article 16-2 discovers a patient of an infectious disease, etc. prescribed by Ordinance of the Ministry of Health and Welfare through a laboratory test, etc., he or she shall report such fact to the head of the relevant institution. <Amended on Jul. 6, 2015; Mar. 27, 2018; Mar. 4, 2020>  (3) Upon receipt of a report under paragraph (1) or (2), the head of a medical institution and the head of an institution for confirming infectious pathogens referred to in Article 16-2 shall report thereon to the Commissioner of the Korea Disease Control and Prevention Agency or the director of the competent public health center, immediately in cases of Class 1 infectious diseases, within 24 hours in cases of Classes 2 and 3 infectious diseases, and within seven days in cases of Class 4 infectious diseases, respectively. <Newly Inserted on Jul. 6, 2015; Mar. 27, 2018; Mar. 4, 2020; Aug. 11, 2020> |
| Article 13 (Reporting by Directors of Public Health Centers) | (1) The director of a public health center in receipt of a report made under Articles 11 and 12 shall report the details thereof to the competent Special Self-Governing City Mayor or Special Self-Governing Province Governor, or the head of the competent Si/Gun/Gu; and the Special Self-Governing City Mayor or the Special Self-Governing Province Governor shall report the same to the Commissioner of the Korea Disease Control and Prevention Agency, and the head of the Si/Gun/Gu to the competent Mayor/Do Governor, respectively. <Amended on Jan. 18, 2010; Aug. 11, 2020; Jun. 13, 2023>  (2) Upon receipt of a report referred to in paragraph (1), the Commissioner of the Korea Disease Control and Prevention Agency, the competent Mayor/Do Governor, or the head of the competent Si/Gun/Gu may require a person falling under Article 11 (1) 4 (limited to persons suspected of contracting any Class 1 infectious disease) to undergo infectious pathogen testing. <Newly Inserted on Mar. 4, 2020; Aug. 11, 2020>  (3) Matters necessary for methods and procedures to report pursuant to paragraph (1), and other relevant matters, shall be determined by Ordinance of the Ministry of Health and Welfare. <Amended on Jan. 18, 2010; Mar. 4, 2020>  [Title Amended on Mar. 4, 2020] |
| Article 14 (Notification of Zoonoses) | (1) Upon receipt of a report referred to in Article 11 (1) 2 of the Act on the Prevention of Contagious Animal Diseases, the head of the national animal disease control agency, the head of a Si/Gun/Gu having jurisdiction over the place where animals subject to reporting are located, or the head of a City/Do animal disease control agency shall immediately notify the Commissioner of the Korea Disease Control and Prevention Agency of contagious animal diseases prescribed in that Act, if they fall under any of the following: <Amended on Dec. 3, 2019; Aug. 11, 2020> |
| Article 16 (Sentinel Surveillance of Infectious Diseases) | (1) The Commissioner of the Korea Disease Control and Prevention Agency may designate a health and medical service institution or any other institution or organization under the Framework Act on Health and Medical Services, as an institution of sentinel surveillance of infectious diseases, in consideration of the characteristics of a disease and the region of the outbreak thereof in order to ensure sentinel surveillance on the outbreak of infectious diseases. <Amended on Jan. 18, 2010; Dec. 3, 2019; Aug. 11, 2020>  (2) The Commissioner of the Korea Disease Control and Prevention Agency, a Mayor/Do Governor, or the head of a Si/Gun/Gu may request the head of an institution of sentinel surveillance of infectious diseases designated under paragraph (1) (hereinafter referred to as "sentinel surveillance institution") to submit necessary data in connection with the sentinel surveillance of infectious diseases, or to provide necessary cooperation for the prevention and control of infectious diseases. In such cases, a sentinel surveillance institution shall comply therewith unless there is a compelling reason not to do so. <Amended on Jan. 18, 2010; Aug. 11, 2020>  (3) The Commissioner of the Korea Disease Control and Prevention Agency, a Mayor/Do Governor, or the head of a Si/Gun/Gu shall provide relevant institutions, organizations, establishments, or citizens with important information on national health collected under paragraph (2). <Amended on Jan. 18, 2010; Aug. 11, 2020>  (4) The Commissioner of the Korea Disease Control and Prevention Agency, a Mayor/Do Governor, or the head of a Si/Gun/Gu may subsidize sentinel surveillance institutions for expenses incurred in sentinel surveillance activities. <Amended on Jan. 18, 2010; Aug. 11, 2020>  (5) The Commissioner of the Korea Disease Control and Prevention Agency may revoke the designation of a sentinel surveillance institution, where it falls under any of the following subparagraphs: <Amended on Jul. 6, 2015; Dec. 3, 2019; Aug. 11, 2020>  1. Where it fails to comply with a request to submit data or provide cooperation referred to in paragraph (2);  2. Where it is unable to conduct sentinel surveillance affairs on infectious diseases due to business closure, etc.;  3. In other cases prescribed by Ordinance of the Ministry of Health and Welfare, including where it is negligent in performing sentinel surveillance affairs on infectious diseases.  (7) If deemed urgently necessary to obtain information related to the likelihood of the outbreak or epidemic of any infectious disease, the Commissioner of the Korea Disease Control and Prevention Agency may request the head of a public institution prescribed by Presidential Decree among public institutions under the Act on the Management of Public Institutions, to provide such information. In such cases, the head of the public institution requested to provide such information shall comply with such request unless there is a compelling reason not to do so. <Amended on Jul. 6, 2015; Aug. 11, 2020> |
| Article 16-2 (Institutions for Confirming Pathogens of Infectious Diseases) | (1) Any of the following institutions (hereinafter referred to as "institution for confirming infectious pathogens") may confirm infectious pathogens through laboratory testing, etc.: <Amended on Aug. 11, 2020; May 19, 2023>  (2) The Commissioner of the Korea Disease Control and Prevention Agency may evaluate and manage the laboratory testing capability of institutions for confirming infectious pathogens to ensure the accuracy and reliability of their testing results of infectious pathogens. <Amended on Aug. 11, 2020>  (3) Matters necessary for the methods and procedures for evaluating and managing the laboratory testing capability of institutions for confirming infectious pathogens referred to in paragraph (2), and other relevant matters shall be prescribed by Ordinance of the Ministry of Health and Welfare.  [This Article Newly Inserted on Mar. 4, 2020] |
| Article 17 (Investigations into Actual Conditions) | (1) The Commissioner of the Korea Disease Control and Prevention Agency and Mayors/Do Governors shall conduct fact-finding surveys to understand the actual conditions of management of and infection by infectious diseases and the actual conditions of resistant bacteria, and publicize the outcomes of such surveys. <Amended on Jan. 18, 2010; Jul. 6, 2015; Dec. 2, 2016; Mar. 4, 2020; Aug. 11, 2020>  (2) With respect to surveys referred to in paragraph (1), the Commissioner of the Korea Disease Control and Prevention Agency or a Mayor/Do Governor may request the heads of relevant institutions, corporations, or organizations, including medical institutions, to submit necessary data or statement of opinions. In such cases, any person in receipt of such request shall comply therewith unless there is good cause. <Newly Inserted on Mar. 4, 2020; Aug. 11, 2020>  (3) Matters necessary for specifics included in fact-finding surveys under paragraph (1); the timing, methods, and procedures for conducting fact-finding surveys and the publication of the outcomes thereof; and other relevant matters, shall be determined by Ordinance of the Ministry of Health and Welfare. <Amended on Jan. 18, 2010; Mar. 4, 2020> |
| Article 18 (Epidemiological Investigations) | (1) Where the Commissioner of the Korea Disease Control and Prevention Agency, a Mayor/Do Governor, or the head of a Si/Gun/Gu deems that an infectious disease breaks out and is likely to be epidemic subsequently or that it is unclear whether a disease is infectious but it is necessary to investigate the cause thereof, he or she shall, without delay, conduct an epidemiological investigation and then provide information concerning the findings thereof to the relevant medical institutions to a necessary extent: Provided, That if necessary for preventing the prevalence of the infectious disease in other areas, such information shall be provided to other medical institutions. <Amended on Jul. 6, 2015; Dec. 3, 2019; Aug. 11, 2020>  (2) The Commissioner of the Korea Disease Control and Prevention Agency, a Mayor/Do Governor, or the head of a Si/Gun/Gu shall establish an epidemiological investigation team to conduct an epidemiological investigation, respectively. <Amended on Aug. 11, 2020>  (3) No one shall commit any of the following acts in the course of an epidemiological investigation conducted by the Commissioner of the Korea Disease Control and Prevention Agency, a Mayor/Do Governor, or the head of a Si/Gun/Gu: <Amended on Jul. 6, 2015; Aug. 11, 2020>  1. Refusing, obstructing, or evading the epidemiological investigation without good cause; 2. Making a false statement or presenting false materials; 3. Intentionally omitting or concealing any fact. |
| Article 18-2 (Request for Epidemiological Investigations) | (1) Where an infectious disease or any disease unknown for its cause has broken out or is likely to break out, medical personnel or the head of a medical institution prescribed in the Medical Service Act may request the Commissioner of the Korea Disease Control and Prevention Agency or a Mayor/Do Governor to conduct an epidemiological investigation under Article 18. <Amended on Aug. 11, 2020>  (2) The Commissioner of the Korea Disease Control and Prevention Agency or a Mayor/Do Governor in receipt of a request prescribed in paragraph (1) shall notify, without delay, the relevant medical personnel or the founder of the relevant medical institution of whether to conduct an epidemiological investigation, the ground therefor, and other relevant matters. <Amended on Aug. 11, 2020>  (3) Matters necessary for requests for conducting an epidemiological investigation under paragraph (1), and the methods, procedures, etc. for notification made under paragraph (2) shall be prescribed by Ordinance of the Ministry of Health and Welfare. [This Article Newly Inserted on Jul. 6, 2015] |
| Article 18-3 (Fosterage of Personnel for Epidemiological Investigations) | (1) The Commissioner of the Korea Disease Control and Prevention Agency may regularly provide education and training on epidemiological investigations to epidemiological investigation officers under Article 60-2 or epidemiological investigation officer trainees. <Amended on Mar. 4, 2020; Aug. 11, 2020; May 19, 2023>  (2) The courses of education and training tailored to each group of epidemiological investigation officers and epidemiological officer trainees under paragraph (1) and other necessary matters shall be prescribed by Ordinance of the Ministry of Health and Welfare. <Amended on May 19, 2023> [This Article Newly Inserted on Jul. 6, 2015] |
| Article 18-4 (Request for Presentation of Materials and Other Relevant Matters) | (1) To efficiently conduct epidemiological investigations, etc. under Article 18, the Commissioner of the Korea Disease Control and Prevention Agency may require the head of a relevant central administrative agency and an institution or organization, etc. prescribed by Presidential Decree to present materials necessary for epidemiological investigations. <Amended on Aug. 11, 2020>  (2) Where a crisis alert of attention level or higher under Article 38 (2) of the Framework Act on the Management of Disasters and Safety is issued in connection with an infectious disease, the Commissioner of the Korea Disease Control and Prevention Agency or a Mayor/Do Governor may request a corporation, organization, individual, etc. to submit materials necessary for epidemiological investigations to efficiently conduct epidemiological investigations under Article 18. <Newly Inserted on May 19, 2023>  (3) Where the Commissioner of the Korea Disease Control and Prevention Agency conducts epidemiological investigations under Article 18, he or she may, if necessary, request the head of a relevant central administrative agency to provide necessary assistance, such as dispatch of the personnel belonging to such agency. <Amended on Aug. 11, 2020; May 19, 2023>  (4) A person in receipt of a request for the presentation of materials under paragraphs (1) and (2) and a request, etc. for assistance under paragraph (3) shall comply therewith, unless there is a compelling reason not to do so. <Amended on May 19, 2023>  (5) Matters necessary for the extent and methods of requests for the presentation of materials under paragraphs (1) and (2), and requests, etc. for assistance under paragraph (3), shall be prescribed by Presidential Decree. <Amended on May 19, 2023> [This Article Newly Inserted on Jul. 6, 2015] |
| Article 18-5 (Provision of Infectious Disease Education) | (1) The heads of State agencies and the heads of local governments shall provide public officials, employees, etc. under their control with education about the prevention and control of infectious diseases and response to crisis (hereinafter referred to as "infectious disease education") at least once a year, and submit the results thereof to the Commissioner of the Korea Disease Control and Prevention Agency.  (2) The head of a public institution under Article 4 of the Act on the Management of Public Institutions may provide infectious disease education to executive officers and employees under his or her jurisdiction.  (3) The Commissioner of the Korea Disease Control and Prevention Agency shall develop and disseminate relevant curriculum to effectively provide infectious disease education under paragraphs (1) and (2).  (4) Matters necessary for the subject, scope, contents, and method of infectious disease education under paragraphs (1) and (2) and the development and dissemination of curriculum under paragraph (3) shall be prescribed by Presidential Decree. [This Article Newly Inserted on Sep. 14, 2023] |
| Article 34 (Formulation and Implementation of Crisis Control Measures against Infectious Diseases) | (1) The Commissioner of the Korea Disease Control and Prevention Agency shall formulate and implement crisis control measures against infectious diseases (hereinafter referred to as "crisis control measures against infectious diseases") after deliberation by the Committee in order to respond to an emergency resulting from the spread of infectious diseases or the transmission of new overseas infectious diseases into the Republic of Korea. <Amended on Jan. 18, 2010; Jul. 6, 2015; Aug. 11, 2020>  (2) Crisis control measures against infectious diseases shall include the following: <Amended on Jan. 18, 2010; Jul. 6, 2015; Aug. 11, 2020; Sep. 29, 2020; Dec. 15, 2020; Mar. 9, 2021; Sep. 14, 2023>  (3) The Commissioner of the Korea Disease Control and Prevention Agency shall regularly conduct training, based on crisis control measures against infectious diseases. <Newly Inserted on Jul. 6, 2015; Aug. 11, 2020>  (4) Matters necessary for the formulation, implementation, etc. of crisis control measures against infectious diseases shall be prescribed by Presidential Decree. <Amended on Jul. 6, 2015> |
| Article 34-2 (Disclosure of Information during Infectious Disease Emergency) | (1) Where the spread of an infectious disease harmful to citizens' health triggers the issuance of a crisis alert of the caution level or higher prescribed in Article 38 (2) of the Framework Act on the Management of Disasters and Safety, the Commissioner of the Korea Disease Control and Prevention Agency, a Mayor/Do Governor, and the head of a Si/Gun/Gu shall promptly disclose information that citizens need to know to prevent the infectious disease, such as the movement paths, transportation means, medical treatment institutions, and contacts of patients of the infectious disease, the current status of the outbreak and testing of the disease infectious by region and by age group, using such means as posting the information on information and communications networks and distributing a press release: Provided, That gender, age, and other information prescribed by Presidential Decree, which is deemed irrelevant to the prevention of the infectious disease, shall be excluded. <Amended on Mar. 4, 2020; Aug. 11, 2020; Sep. 29, 2020; Mar. 9, 2021>  (2) The Commissioner of the Korea Disease Control and Prevention Agency, a Mayor/Do Governor, or the head of a Si/Gun/Gu shall delete the information disclosed pursuant to paragraph (1) without delay if it is no longer necessary to disclose such information due to the achievement of the purpose of disclosure, etc. <Newly Inserted on Sep. 29, 2020>  (3) Where any information disclosed under paragraph (1) falls under any of the following subparagraphs, the relevant person may file an objection with the Commissioner of the Korea Disease Control and Prevention Agency, a Mayor/Do Governor, or the head of a Si/Gun/Gu in writing, orally, or using information and communications networks: <Newly Inserted on Mar. 4, 2020; Aug. 11, 2020; Sep. 29, 2020> 1. Where any disclosed information is different from the actual fact; 2. Where he or she has any opinion on any disclosed information.  (4) Where the Commissioner of the Korea Disease Control and Prevention Agency deems that the objection raised under paragraph (3) is well-grounded, he or she shall without delay take necessary measures, such as correcting the relevant disclosed information. <Newly Inserted on Mar. 4, 2020; Aug. 11, 2020; Sep. 29, 2020>  (5) Matters necessary for disclosing and deleting information and the scope of, procedures, methods, etc. for raising objections under paragraphs (1) through (3) shall be prescribed by Ordinance of the Ministry of Health and Welfare. <Amended on Mar. 4, 2020; Sep. 29, 2020> [This Article Newly Inserted on Jul. 6, 2015] |
| Article 35 (Formulation of Crisis Control Measures against Infectious Diseases by City/Do) | (1) The Commissioner of the Korea Disease Control and Prevention Agency shall notify Mayors/Do Governors of crisis control measures against infectious diseases formulated under Article 34 (1). <Amended on Jan. 18, 2010; Aug. 11, 2020>  (2) Each Mayor/Do Governor shall formulate and implement crisis control measures against infectious diseases by each Special Metropolitan City, Metropolitan City, Special Self-Governing City, Do, or Special Self-Governing Province (hereinafter referred to as "City/Do"), based on the crisis control measures against infectious diseases notified under paragraph (1). <Amended on Jun. 13, 2023> |
| Article 43-2 (Notification to Persons Subject to Quarantine or Isolation) | (1) Where the Commissioner of the Korea Disease Control and Prevention Agency, a Mayor/Do Governor, or the head of a Si/Gun/Gu takes any measure for hospitalization, quarantine, or isolation prescribed in Article 42 (2), (3), and (7), subparagraph 3 of Article 47, or Article 49 (1) 14, he or she shall notify such fact to persons subject to hospitalization, quarantine, or isolation and his or her guardian. <Amended on Aug. 11, 2020>  (2) Matters necessary for the methods of, procedures, etc. for notification referred to in paragraph (1) shall be prescribed by Ordinance of the Ministry of Health and Welfare. [This Article Newly Inserted on Mar. 4, 2020] |
| Article 49 (Preventive Measures against Infectious Diseases) | (1) In order to prevent infectious diseases, the Commissioner of the Korea Disease Control and Prevention Agency, Mayors/Do Governors, or heads of Sis/Guns/Gus shall take all or some of the following measures; and the Minister of Health and Welfare may take measures under subparagraphs 2, 2-2 through 2-4, 12, and 12-2 to prevent infectious diseases: <Amended on Jul. 6, 2015; Dec. 29, 2015; Mar. 4, 2020; Aug. 11, 2020; Aug. 12, 2020; Sep. 29, 2020; Mar. 9, 2021>  2-2. Ordering the managers, operators, users, etc. of places or facilities with a risk of spreading an infectious disease to comply with the disease control guidelines, such as preparing a list of visitors and wearing a mask;  2-3. Ordering users of means of transport likely to transmit infectious diseases, such as buses, trains, ships, and aircraft to comply with disease control guidelines, such as wearing a mask; |
| Article 60 (Disease Control Officers) | (1) The Commissioner of the Korea Disease Control and Prevention Agency and each Mayor/Do Governor shall appoint disease control officers in charge of the affairs of infectious disease prevention and control, from among public officials of said Ministry or City/Do: Provided, That if necessary for dealing with the affairs of infectious disease prevention and control, the head of a Si/Gun/Gu may appoint disease control officers from among public officials of said Si/Gun/Gu. <Amended on Mar. 4, 2020; Aug. 11, 2020>  (2) Each disease control officer shall be in charge of affairs specified in Article 4 (2) 1 through 7: Provided, That each disease control officer of the Korea Disease Control and Prevention Agency shall also be in charge of affairs specified in Article 4 (2) 8. <Amended on Aug. 11, 2020>  4) Relevant public officials, such as the head of a police agency prescribed in Articles 12 and 13 of the Act on the Organization and Operation of National Police and Autonomous Police, the head of a fire-fighting government office prescribed in Article 3 of the Framework Act on Firefighting Services, and the director of a public health center prescribed in Article 10 of the Regional Public Health Act, all of which have jurisdiction over an area of an infectious disease, and corporations, organizations, and individuals located in that area shall cooperate in measures taken by a disease control officer under paragraph (3) without good cause. <Amended on Dec. 22, 2020> |
| Article 60-2 (Epidemiological Investigation Officers) | (1) Epidemiological investigation officers shall be composed of at least 100 public officials of the Korea Disease Control and Prevention Agency and at least two public officials of a City/Do, respectively, to deal with affairs concerning epidemiological investigations. In such cases, at least one of the City/Do epidemiological investigation officers shall be a physician, among the medical personnel referred to in Article 2 (1) of the Medical Service Act. <Amended on Mar. 27, 2018; Mar. 4, 2020; Aug. 11, 2020>  (2) Where necessary for dealing with affairs concerning epidemiological investigations, the head of a Si/Gun/Gu may have epidemiological investigation officers as public officials of said Si/Gun/Gu: Provided, That the head of a Si/Gun/Gu that meets the criteria prescribed by Ordinance of the Ministry of Health and Welfare in consideration of the population, etc. shall have at least one epidemiological investigation officer as a public official of said Si/Gun/Gu. <Newly Inserted on Mar. 4, 2020>  (3) Epidemiological investigation officers under paragraphs (1) and (2) shall be appointed, from among any of the following persons who have completed education and training courses on epidemiological investigations under Article 18-3: <Amended on Mar. 4, 2020; May 19, 2023>  (4) Where the Commissioner of the Korea Disease Control and Prevention Agency, a Mayor/Do Governor, or the head of a Si/Gun/Gu needs to require a public official under his or her control to complete education and training courses on epidemiological investigations under Article 18-3 so as to appoint the public official as an epidemiological investigation officer, he or she shall appoint the relevant public official as an epidemiological investigation officer trainee. <Newly Inserted on May 19, 2023>  (5) An epidemiological investigation officer may temporarily take the measures specified under the items of subparagraph 1 of Article 47, where an emergency, in which the spread of an infectious disease is anticipated, would be likely to cause a serious harm to public health if measures thereagainst are not taken immediately: Provided, That epidemiological investigation officer trainees may temporarily take the measures under the items of subparagraph 1 of Article 47, only if under the command of disease control officers or epidemiological investigation officers. <Amended on Mar. 4, 2020; May 19, 2023>  (6) Relevant public officials, such as the head of a police agency under Articles 12 and 13 of the Act on the Organization and Operation of National Police and Autonomous Police, the head of a firefighting government office under Article 3 of the Framework Act on Firefighting Services, and the director of a public health center under Article 10 of the Regional Public Health Act, shall cooperate in measures taken by an epidemiological investigation officer or an epidemiological investigation officer trainee under paragraph (5) without good cause. <Amended on Mar. 4, 2020; Dec. 22, 2020; May 19, 2023> (7) Where an epidemiological investigation officer or an epidemiological investigation officer trainee takes the measures under paragraph (5), he or she shall immediately report such fact to the Commissioner of the Korea Disease Control and Prevention Agency, the competent Mayor/Do Governor, or the head of the competent Si/Gun/Gu. <Amended on Mar. 4, 2020; Aug. 11, 2020; May 19, 2023> (8) The Commissioner of the Korea Disease Control and Prevention Agency, a Mayor/Do Governor, or the head of a Si/Gun/Gu may subsidize epidemiological investigation officers and epidemiological investigation officer trainees appointed pursuant to paragraphs (1), (2) and (4) for expenses necessary to perform their duties, etc., within the budget. <Amended on Mar. 4, 2020; Aug. 11, 2020; May 19, 2023> (9) Except as provided in paragraphs (1) through (8), matters necessary for the qualification, duties, authority of epidemiological investigation officers and epidemiological investigation officer trainees, subsidization therefor, etc. shall be prescribed by Presidential Decree. <Amended on Mar. 4, 2020; May 19, 2023> [This Article Newly Inserted on Jul. 6, 2015] |
| Article 67 (Expenses to Be Borne by National Treasury) | The following expenses shall be borne by the National Treasury: <Amended on Jan. 18, 2010; Jul. 6, 2015; Dec. 29, 2015; Mar. 27, 2018; Dec. 3, 2019; Mar. 4, 2020; Aug. 11, 2020; Aug. 12, 2020; Sep. 29, 2020; Dec. 15, 2020; Sep. 14, 2023>  1. Expenses incurred in the medical treatment and protection of patients of an infectious disease, etc. under Article 4 (2) 2; 2. Expenses incurred in the education and publicity of infectious diseases under Article 4 (2) 4; 3. Expenses incurred in nurturing specialists for the prevention of infectious diseases under Article 4 (2) 8; 4. Expenses incurred in conducting sentinel surveillance activities under Article 16 (4); 4-2. Expenses incurred in conducting education and training under Article 18-3; 5. Expenses incurred in transporting corpses for autopsies under Article 20, and in disposal of them after autopsies; 5-2. Expenses incurred in conducting funerals for the deceased persons under Article 20-2; 6. Expenses incurred in the production, research, etc., of vaccines under Article 33; 6-2. Expenses incurred in stockpiling mandatory vaccines, etc. under Article 33-2 (1); 6-3. Expenses incurred in the State's crisis response training under Article 34 (2) 5; |
| Article 76-2 (Request for Provision of Information and Verification of Information) | (1) If necessary to prevent and control infectious diseases and block the spread of infection, the Commissioner of the Korea Disease Control and Prevention Agency or a Mayor/Do Governor may request the heads of relevant central administrative agencies (including affiliated agencies and responsible administrative agencies thereof), the heads of local governments (including the superintendents of education prescribed in Article 18 of the Local Education Autonomy Act), public institutions designated under Article 4 of the Act on the Management of Public Institutions, medical institutions, pharmacies, corporations, organizations, and individuals to provide the following information concerning patients of infectious diseases, etc., persons suspected of contracting an infectious disease, and persons vaccinated, and persons in receipt of such request shall comply therewith: <Amended on Dec. 2, 2016; Mar. 4, 2020; Aug. 11, 2020; Sep. 29, 2020; Mar. 28, 2023> 1. Personal information, such as names, resident registration numbers prescribed in Article 7-2 (1) of the Resident Registration Act, addresses, and telephone numbers (including cell phone numbers); 2. Prescriptions prescribed in Article 17 of the Medical Service Act and medical records, etc. prescribed in Article 22 of that Act; 3. Information prescribed by Presidential Decree as necessary for preventing and controlling infectious diseases, such as severity levels of disability, types of disability, and income distribution, among information about the insured and their dependents under Article 5 of the National Health Insurance Act or eligible recipients under Article 3 of the Medical Benefit Act; 4. Information, such as medical records, medicines, injuries and diseases, prescribed by Presidential Decree, regarding claims for and payment of costs of health care benefits under Article 47 of the National Health Insurance Act, and regarding claims for and granting of expenses for benefits under Article 11 of the Medical Benefit Act; 5. Records of immigration control during the period determined by the Commissioner of the Korea Disease Control and Prevention Agency; 6. Other information prescribed by Presidential Decree for monitoring the movement paths of such patients, etc.  (2) If necessary to prevent and control infectious diseases and block the spread of infection, the Commissioner of the Korea Disease Control and Prevention Agency, a Mayor/Do Governor, or the head of a Si/Gun/Gu may request the Commissioner General of the Korean National Police Agency, the commissioner of a City/Do police agency, or the chief of a police station referred to in Article 2 of the Act on the Organization and Operation of National Police and Autonomous Police (hereafter in this Article referred to as "police agency") to provide location information of patients of an infectious disease, etc. and persons suspected of contracting an infectious disease. In such cases, notwithstanding Article 15 of the Act on the Protection and Use of Location Information and Article 3 of the Protection of Communications Secrets Act, the head of the relevant police agency, upon request by the Commissioner of the Korea Disease Control and Prevention Agency, a Mayor/Do Governor, or the head of a Si/Gun/Gu, may request any personal location information provider defined in Article 5 (7) of the Act on the Protection and Use of Location Information and any telecommunications business operator defined in subparagraph 8 of Article 2 of the Telecommunications Business Act to provide location information of patients of an infectious disease, etc. and persons suspected of contracting an infectious disease; and the personal location information provider and the telecommunications business operator in receipt of such request shall comply therewith unless there is good cause. <Amended on Dec. 29, 2015; Apr. 17, 2018; Mar. 4, 2020; Aug. 11, 2020; Dec. 22, 2020; Mar. 28, 2023>  (3) The Commissioner of the Korea Disease Control and Prevention Agency may provide information collected pursuant to paragraphs (1) and (2) to the heads of the relevant central administrative agencies, the heads of local governments, the President of the National Health Insurance Service, the President of the Health Insurance Review and Assessment Service, health and medical services institutions defined in subparagraph 4 of Article 3 of the Framework Act on Health and Medical Services (hereinafter referred to as "health and medical services institutions"), other organizations, etc. In such cases, information provided to health and medical services institutions, etc. shall be limited to information related to the affairs of the relevant institutions, etc. for preventing and controlling infectious diseases and blocking the spread of infection. <Amended on Mar. 4, 2020; Aug. 11, 2020; Mar. 28, 2023>  (4) Notwithstanding the former part of paragraph (3), if necessary to prevent and control infectious diseases and block the spread of infection, the Commissioner of the Korea Disease Control and Prevention Agency shall provide information prescribed in paragraph (1) 5 and information on movement paths prescribed in subparagraph 6 of that paragraph to health and medical services institutions using any of the following information and communications systems. In such cases, information provided to health and medical services institutions shall be limited to information related to the affairs of the relevant institutions: <Newly Inserted on Mar. 4, 2020; Aug. 11, 2020; Mar. 28, 2023>  1. The information system of the National Health Insurance Service; 2. The information system of the Health Insurance Review and Assessment Service; 3. The information system of an institution deemed necessary and designated by the Commissioner of the Korea Disease Control and Prevention Agency to prevent any infectious disease from being transmitted into or spreading in the Republic of Korea.  (5) When providing medical treatment or prescribing or preparing medicines, medical personnel, pharmacists, and the heads of health and medical services institutions shall check information provided pursuant to paragraph (4) using an information system prescribed in any subparagraph of that paragraph. <Newly Inserted on Mar. 4, 2020>  (6) No person provided with information pursuant to paragraphs (3) and (4) shall use such information for any purpose, other than conducting affairs related to infectious diseases under this Act, and shall, without delay, destroy all information after completing the relevant affairs and inform the Commissioner of the Korea Disease Control and Prevention Agency thereof. <Amended on Mar. 4, 2020; Aug. 11, 2020>  (7) The Commissioner of the Korea Disease Control and Prevention Agency, a Mayor/Do Governor, or the head of a Si/Gun/Gu shall notify the subject of information collected pursuant to paragraphs (1) and (2) (hereinafter referred to as “data subject”), of the following facts: <Amended on Mar. 4, 2020; Aug. 11, 2020; Mar. 28, 2023; Jan. 23, 2024>  1. The fact that information necessary for preventing and controlling infectious diseases and blocking the spread of infection has been collected; 2. Where information prescribed in subparagraph 1 has been provided to another agency, such fact; 3. The fact that, even in cases prescribed in subparagraph 2, no information shall be used for any purpose, other than conducting affairs related to infectious diseases under this Act, and all the information shall be destroyed without delay when the relevant affairs are completed.  (8) Where a person provided with information pursuant to paragraphs (3) and (4) processes the relevant information in violation of this Act, such person shall be governed by the Personal Information Protection Act. <Amended on Mar. 4, 2020>  (9) Matters necessary for the target and scope of information provided under paragraph (3), the methods of notification under paragraph (7), and other relevant matters shall be prescribed by Ordinance of the Ministry of Health and Welfare. <Amended on Mar. 4, 2020> [This Article Newly Inserted on Jul. 6, 2015] [Title Amended on Mar. 4, 2020] |
| Article 76-3 (Preparation and Disclosure of Personal Information Processing Report) | (1) The Commissioner of the Korea Disease Control and Prevention Agency shall prepare a report on the processing of personal information (hereinafter referred to as "personal information processing report"), including notification to data subjects under Article 76-2 (7), every year.  (2) The Mayor/Do Governor or the head of a Si/Gun/Gu shall submit data related to notification to data subjects under Article 76-2 (7) to the Commissioner of the Korea Disease Control and Prevention Agency.  (3) The Commissioner of the Korea Disease Control and Prevention Agency shall disclose a personal information processing report prepared pursuant to paragraph (1) on the website of the Korea Disease Control and Prevention Agency by the first half of the following year, as prescribed by Ordinance of the Ministry of Health and Welfare.  (4) Matters necessary for the preparation of personal information processing reports and submission of data shall be prescribed by Ordinance of the Ministry of Health and Welfare. [This Article Newly Inserted on Jan. 23, 2024] [Previous Article 76-3 moved to Article 76-4 <Jan. 23, 2024>] |
|  |  |

Table S3. Medical Response System Enhancement

Title: INFECTIOUS DISEASE CONTROL AND PREVENTION ACT

| ARTICLE | CONTENT |
| --- | --- |
| Article 5 (Responsibilities and Rights of Medical Personnel) | (2) Medical personnel, the heads of medical institutions, etc. prescribed in the Medical Service Act shall make utmost effort for the diagnosis, management, treatment, etc., of patients of infectious diseases, and shall actively cooperate to comply with administrative orders issued by the Minister of Health and Welfare, the Commissioner of the Korea Disease Control and Prevention Agency, or the heads of local governments.  <Amended on Aug. 11, 2020> |
| Article 8-2 (Infectious Disease Hospitals) | (1) The State shall establish, or operate by designation, the Central Infectious Disease Hospital, equipped with adequate facilities, personnel, and research capabilities to pursue research and prevention of infectious diseases, to nurture and train infectious disease specialists, and to diagnose and treat patients of infectious diseases. <Amended on Aug. 16, 2023>  (2) For the medical examination and treatment of patients with infectious diseases, the State shall establish or designate and operate regional specialized hospitals for infectious diseases in each region, which are equipped with the number of sickbeds (including negative pressure isolation rooms and isolation beds) in a scale not below the standards prescribed by Ordinance of the Ministry of Health and Welfare. In such cases, the State shall set up the region in consideration of the population, geographical accessibility, etc. <Amended on Oct. 19, 2021; Aug. 16, 2023>  (3) The State may provide budget support for establishing, or operating by designation, the Central Infectious Disease Hospital or a regional infectious disease hospital under paragraph (1) or (2), within the budget. <Amended on Aug. 16, 2023> |
| Article 8-2 (Infectious Disease Hospitals) | (4) The State may establish the Clinical Committee for Infectious Diseases at the Central Infectious Disease Hospital under paragraph (1) in order to provide advice, etc. on the business affairs of the Central Infectious Disease Hospital. <Newly Inserted on Aug. 16, 2023>  (5) Procedures necessary for and methods of establishing, or operating by designation, the Central Infectious Disease Hospital or a regional infectious disease hospital under paragraph (1) or (2), and details of support therefor shall be prescribed by Presidential Decree. <Amended on Aug. 16, 2023> [This Article Newly Inserted on Dec. 29, 2015] |
| Article 41 (Control of Patients with Infectious Disease) | (1) Patients of an infectious disease, etc. with a particularly high risk of transmission, which falls under Class 1 infectious diseases or is an infectious disease publicly notified by the Commissioner of the Korea Disease Control and Prevention Agency, shall receive inpatient treatment at an infectious disease control institution, the Central Infectious Disease Hospital, a regional infectious disease hospital, or a medical institution with infectious disease control facilities (hereinafter referred to as "infectious disease control institution, etc."). <Amended on Jan. 18, 2010; Mar. 27, 2018; Aug. 11, 2020; Aug. 12, 2020; Aug. 16, 2023>  (2) The Commissioner of the Korea Disease Control and Prevention Agency, a Mayor/Do Governor, or the head of a Si/Gun/Gu may allow any of the following persons to undergo self-care, treatment at facilities established and operated under Article 37 (1) 2 (hereinafter referred to as "facility treatment"), or inpatient treatment at medical institutions: <Amended on Jan. 18, 2010; Aug. 11, 2020; Aug. 12, 2020>  1. A person deemed by a medical doctor to be capable of undergoing self-care or facility treatment, notwithstanding paragraph (1);  2. A person who is not subject to inpatient treatment under paragraph (1);  3. A person suspected of contracting an infectious disease.  (3) In any of the following cases, the Minister of Health and Welfare, the Commissioner of the Korea Disease Control and Prevention Agency, a Mayor/Do Governor or the head of a Si/Gun/Gu may transfer persons undergoing treatment under paragraph (1) or (2) to other infectious disease control institutions or medical institutions which are not an infectious disease control institution, allow them to undergo self-care, or relocate them to facilities established and operated under Article 37 (1) 2 (hereinafter referred to as "transfer, etc."): <Newly Inserted on Aug. 12, 2020; Sep. 29, 2020>  1. Where there is a change in severity;  2. Where the doctor deems that hospitalization is unnecessary;  3. Where the Commissioner of the Korea Disease Control and Prevention Agency deems that transfer, etc. is necessary due to shortage of isolation beds, etc.  (4) Patients, etc. with an infectious disease shall comply with the measures under paragraph (3), and where they refuse it without good cause, they shall bear expenses incurred in relation to medical treatment. <Newly Inserted on Aug. 12, 2020>  (5) Matters necessary regarding methods and procedures of inpatient treatment, self-care, and facility treatment under paragraphs (1) and (2) and methods and procedures of transfer, etc. under paragraph (3) shall be prescribed by Presidential Decree. <Amended on Aug. 12, 2020> |
| Article 42 (Compulsory Dispositions with respect to Infectious Diseases) | (2) Where any Class 1 infectious disease breaks out, the Commissioner of the Korea Disease Control and Prevention Agency, a Mayor/Do Governor, or the head of a Si/Gun/Gu may have the relevant public official take the following measures for persons suspected of contracting the infectious disease. In such cases, the relevant public official may conduct a necessary investigation or medical diagnosis to confirm the presence or absence of infectious disease symptoms: <Newly Inserted on Mar. 4, 2020; Aug. 11, 2020; Sep. 29, 2020> (3) With respect to persons deemed patients of an infectious disease, etc. as a result of investigations or medical diagnosis referred to in paragraph (2), the Commissioner of the Korea Disease Control and Prevention Agency, a Mayor/Do Governor, or the head of a Si/Gun/Gu may assign the relevant public official to escort and compel such persons to undergo medical treatment or be hospitalized. <Newly Inserted on Mar. 4, 2020; Aug. 11, 2020>  (4) Where a person refuses an investigation or medical diagnosis referred to in paragraphs (1) and (2) or a test referred to in Article 13 (2) (hereafter in this Article referred to as "investigation refuser"), the Commissioner of the Korea Disease Control and Prevention Agency, a Mayor/Do Governor, or the head of a Si/Gun/Gu shall assign the relevant public official to escort such person to an infectious disease control institution and compel such person to undergo necessary investigation or diagnosis. <Amended on Dec. 29, 2015; Mar. 4, 2020; Aug. 11, 2020>  (5) A public official who takes measures for investigation, medical diagnosis, quarantine or isolation, treatment, hospitalization, or escort pursuant to paragraphs (1) through (4) shall carry an identification indicating his or her authority and produce it to relevant persons. <Newly Inserted on Dec. 29, 2015; Mar. 4, 2020>  (6) Where necessary for taking any measure for investigation, medical diagnosis, quarantine or isolation, treatment, or hospitalization prescribed in paragraphs (2) through (4) and (7), the Commissioner of the Korea Disease Control and Prevention Agency, a Mayor/Do Governor, or the head of a Si/Gun/Gu may request cooperation from the chief of the competent police station. In such cases, the chief of the competent police station in receipt of such request shall comply therewith unless there is a compelling reason not to do so. <Newly Inserted on Dec. 29, 2015; Mar. 4, 2020; Aug. 11, 2020>  (7) The Commissioner of the Korea Disease Control and Prevention Agency, a Mayor/Do Governor, or the head of a Si/Gun/Gu may quarantine or isolate any investigation refuser at such refuser’s home or in an infectious disease control facility; and if the investigation refuser is deemed a patient of an infectious disease, etc. according to the results of an investigation or medical diagnosis conducted under paragraph (4), he or she shall compel such patient to undergo medical treatment or to be hospitalized in an infectious disease control facility. <Newly Inserted on Dec. 29, 2015; Mar. 4, 2020; Aug. 11, 2020>  (8) Where any person suspected of contracting an infectious disease or investigation refuser is found not to be a patient of an infectious disease, etc., the Commissioner of the Korea Disease Control and Prevention Agency, a Mayor/Do Governor, or the head of a Si/Gun/Gu shall immediately release the investigator refuser from quarantine or isolation referred to in paragraph (2) or (7). <Newly Inserted on Dec. 29, 2015; Mar. 4, 2020; Aug. 11, 2020>  (9) Where the Commissioner of the Korea Disease Control and Prevention Agency, a Mayor/Do Governor, or the head of a Si/Gun/Gu gives medical treatment to, or hospitalizes, any investigation refuser pursuant to paragraph (7), he or she shall notify the guardian of the investigation refuser thereof. In such cases, Article 43 shall apply mutatis mutandis to matters necessary for the methods of, procedures, etc. for notification. <Newly Inserted on Dec. 29, 2015; Mar. 4, 2020; Aug. 11, 2020>  (10) Notwithstanding paragraph (8), if a disposition of quarantine or isolation is not released without good cause, the relevant persons suspected of contracting an infectious disease or investigation refuser may make a rescue claim seeking the release; and in regards to the process, methods, etc. of such rescue claim, the Habeas Corpus Act shall apply mutatis mutandis. In such cases, "person suspected of contracting an infectious disease or investigation refuser" shall be construed as "inmate"; and "Commissioner of the Korea Disease Control and Prevention Agency, a Mayor/Do Governor, or the head of a Si/Gun/ Gu" who has ordered the disposition of quarantine or isolation shall be construed as "custodian" (for the purposes of this paragraph, the application of Article 6 (1) 3 of the Habeas Corpus Act shall be excluded). <Newly Inserted on Dec. 29, 2015; Mar. 4, 2020; Aug. 11, 2020>  (11) Matters necessary for the criteria for designating institutions to conduct investigations, medical diagnosis, quarantine or isolation, or treatment under paragraphs (1) through (4) and (7), the methods for quarantine and for checking the presence or absence of symptoms with regard to persons suspected of contracting an infectious disease under paragraph (2), and other relevant matters shall be prescribed by Presidential Decree. <Newly Inserted on Dec. 29, 2015; Mar. 4, 2020>  (12) Matters concerning the storage, protection, use, and destruction of location information collected pursuant to paragraph (2) 2 shall be governed by the Act on the Protection and Use of Location Information. <Newly Inserted on Sep. 29, 2020> |
| Article 49 (Preventive Measures against Infectious Diseases) | 12-2. Mobilizing of facilities, such as sickbeds of medical institutions, training institutes, and accommodation facilities, during the epidemic period of an infectious disease; (3) A Mayor/Do Governor or the head of a Si/Gun/Gu may order a manager or operator who fails to comply with the measures under paragraph (1) 2-2 to close the relevant place or facility, or to suspend operation within a prescribed period not exceeding three months: Provided, That where a person who has received an order to suspend operation continues to operate the relevant place or facility during the period of suspension of operation, he or she shall issue an order requiring closure of the relevant place or facility. <Newly Inserted on Sep. 29, 2020; Mar. 9, 2021>  (4) A manager or operator in receipt of an order to close a place or facility or to suspend the operation thereof under paragraph (3) shall comply therewith unless there is good cause. <Newly Inserted on Mar. 9, 2021>  (5) Where a manager or operator continues to operate a facility despite the closure order issued under paragraph (3), the Mayor/Do Governor or the head of the Si/Gun/Gu may require the relevant public official to take the following measures to close the relevant place or facility: <Newly Inserted on Sep. 29, 2020; Mar. 9, 2021>  1. Removing the signboard or other signs of the relevant place or facility;  2. Posting a notice informing that the relevant place or facility is closed under paragraph (3).  (6) A Mayor/Do Governor or the head of a Si/Gun/Gu who has ordered the closing a place or facility pursuant to paragraph (3) may determine whether to suspend the closing of the relevant place or facility subject to deliberation by a local committee under Article 11 of the Framework Act on the Management of Disasters and Safety, if it is no longer necessary to close the relevant place or facility due to a change in crisis alert or guidelines for disease control. <Newly Inserted on Mar. 9, 2021>  (5) Criteria for administrative dispositions referred to in paragraph (3) shall be prescribed by Ordinance of the Ministry of Health and Welfare, taking into account the type, severity, etc. of the relevant violation. <Newly Inserted on Sep. 29, 2020; Mar. 9, 2021> |
| Article 49-3 (Temporary Non-Face-to-Face Diagnosis to Protect Medical Personnel, Patients, and Medical Institutions) | (1) When a crisis alert of a serious level or higher is issued under Article 38 (2) of the Framework Act on the Management of Disasters and Safety, notwithstanding Article 33 (1) of the Medical Service Act, medical personnel (limited to medical doctors, dentists, and oriental medical doctors among medical personnel defined in Article 2 of the Medical Service Act; hereafter in this Article, the same shall apply) engaged in medical service may continuously observe, diagnose, consult with, and prescribe medicines for, patients outside medical institutions with respect to their health and disease by using information and communications technologies such as wired, wireless, or image communications, and computers, within the scope determined by the Minister of Health and Welfare, where deemed necessary to protect patients, medical personnel, medical institutions, and others from the risk of infection.  (2) The Minister of Health and Welfare shall determine the scope, such as areas and period of temporary non-face-to-face diagnosis under paragraph (1), subject to deliberation by the Committee. [This Article Newly Inserted on Dec. 15, 2020] |
| Article 69-2 (Expenses Borne by Foreigners) | The Commissioner of the Korea Disease Control and Prevention Agency may require a patient, etc. with an infectious disease who is a foreigner or a person suspected of contracting an infectious disease to bear all or part of the following expenses, taking into account international practices, the principle of reciprocity, etc.: Provided, That the foregoing shall not apply to foreigners confirmed to have been infected with an infectious disease in Korea:  1. Expenses for treatment under Article 41; 2. Expenses for investigation, diagnosis, treatment, hospitalization and quarantine under Article 42. [This Article Newly Inserted on Aug. 12, 2020] |
| Article 70-3 (Financial Support for Health and Medical Services Personnel) | (1) The Commissioner of the Korea Disease Control and Prevention Agency, a Mayor/Do Governor, and the head of a Si/Gun/Gu may subsidize the medical persons, founders of medical institutions, or pharmacists who have supported activities for the surveillance, prevention, control, or epidemiological investigation of an infectious disease under this Act, within the budget. <Amended on Aug. 11, 2020; Dec. 15, 2020>  (2) Where a crisis alert of serious level or higher is issued under Article 38 (2) of the Framework Act on the Management of Disasters and Safety due to the spread of infectious diseases, the Commissioner of the Korea Disease Control and Prevention Agency, a Mayor/Do Governor, or the head of a Si/Gun/Gu may provide financial support to the health and medical services personnel working in health and medical institutions (referring to the health and medical services personnel defined in subparagraph 3 of Article 2 of the Act on Support for Health and Medical Services Personnel and the staff working in health and medical institutions defined in 4 of that Article) who provide assistance to the affairs related to monitoring the outbreak of infectious diseases, and the prevention, quarantine, examination, treatment, management, and epidemiological investigation of the infectious disease cases pursuant to this Act, within the budget. <Newly Inserted on Dec. 21, 2021>  (3) Matters necessary for the details, procedures, methods, etc. of support under paragraphs (1) and (2) shall be prescribed by Presidential Decree. <Amended on Dec. 21, 2021> [This Article Newly Inserted on Dec. 29, 2015] [Title Amended on Dec. 15, 2020; Dec. 21, 2021] |
| Article 70-6 (Psychological Support) | (1) The Minister of Health and Welfare, a Mayor/Do Governor, or the head of a Si/Gun/Gu may provide psychological support defined in Article 15-2 of the Act on the Improvement of Mental Health and the Support for Welfare Services for Mental Patients (hereinafter referred to as "psychological support") to patients of an infectious disease, etc., their families, persons suspected of contracting an infectious disease, medical personnel responding to infectious diseases, and other on-site response personnel.  (2) The Minister of Health and Welfare, a Mayor/Do Governor, or the head of a Si/Gun/Gu may delegate or entrust psychological support to the National Trauma Center under Article 15-2 of the Act on the Improvement of Mental Health and the Support for Welfare Services for Mental Patients or a specialized institution prescribed by Presidential Decree.  (3) Matters necessary for the scope of on-site response personnel under paragraph (1) and psychological support under paragraph (2) shall be prescribed by Presidential Decree. [This Article Newly Inserted on Sep. 29, 2020] |
| Article 79-3 (Penalty Provisions) | Any of the following persons shall be punished by imprisonment with labor for not more than one year or by a fine not exceeding 20 million won: <Amended on Dec. 3, 2019; Sep. 29, 2020; May 19, 2023>  1. Upon receipt of a request for submission of data by the Commissioner of the Korea Disease Control and Prevention Agency or a Mayor/Do Governor under Article 18-4 (2), a person who refuses, obstructs, or evades such requests, who submits false data, or who intentionally omits or conceals any fact, in violation of Article 18-4 (4); 2. A person who handles high-risk pathogens, in violation of Article 23-4 (1); 3. A person who has another person handle high-risk pathogens, in violation of Article 23-4 (2); 4. A medical institution or pharmacy, corporation, organization, or individual that refuses to comply with a request by the Commissioner of the Korea Disease Control and Prevention Agency or a Mayor/Do Governor or that provides false data, in violation of Article 76-2 (1); 5. A person who refuses to comply with a request made by the head of a police agency or provides false data, in violation of the latter part of Article 76-2 (2). [This Article Newly Inserted on Dec. 29, 2015] |

Table S4. Medical Response System Enhancement: Vaccination Program Development

Title: INFECTIOUS DISEASE CONTROL AND PREVENTION ACT

| ARTICLE | CONTENT |
| --- | --- |
| Article 33-4 (Establishment and Operation of Integrated Vaccination Management System) | (1) To efficiently process various data or information required for providing vaccination services and computerize the recording and management affairs, the Commissioner of the Korea Disease Control and Prevention Agency shall establish and operate an integrated vaccination management system (hereinafter referred to as the "Integrated Management System"). <Amended on Aug. 11, 2020>  (2) The Commissioner of the Korea Disease Control and Prevention Agency may collect, manage, and maintain the following data for establishing and operating the Integrated Management System, and request related agencies and organizations to provide necessary data. In such cases, the agencies and organizations in receipt of such request shall comply therewith unless there is good cause: <Amended on Aug. 11, 2020; Mar. 28, 2023>  1. Personal information of persons who should be vaccinated (including personally identifiable information referred to in Article 24 of the Personal Information Protection Act and other personal information prescribed in Presidential Decree);  2. Details of vaccinations, including the names of persons vaccinated, vaccine names, and dates of vaccinations;  3. Other data prescribed by Presidential Decree as necessary for vaccination services, including information on the opening of medical institutions entrusted with vaccination services, details of reporting on adverse reactions to vaccinations under Articles 11 and 13, details of epidemiological investigations on vaccination under Article 29, details of applications for compensation for injuries caused by vaccination under Article 71.  (3) The director of each public health center and the head of each medical institution entrusted with vaccination services under Article 24 (2) (including where the same is applied mutatis mutandis in Article 25 (2)), after providing vaccination services under this Act, shall enter information specified in paragraph (2) 2 in the Integrated Management System, as prescribed by Presidential Decree.  (4) The Commissioner of the Korea Disease Control and Prevention Agency may provide the parents of children who should be vaccinated with the details of vaccinations of their children or may support the issuance of certificates of vaccination, by utilizing the Integrated Management System, as prescribed by Presidential Decree. In such cases, to verify suitability for providing the details of vaccinations or issuing a certificate of vaccination, he or she may request the Minister of National Court Administration to furnish computerized registration data referred to in Article 11 of the Act on the Registration of Family Relations; and the Minister of National Court Administration shall comply therewith unless there is good cause. <Amended on Aug. 11, 2020>  (5) The Integrated Management System may be utilized through electronic linkage with the following information systems related to vaccination services: <Amended on Jan. 11, 2022>  1. The education information system referred to in Article 30-4 of the Elementary and Secondary Education Act;  2. The early childhood education information system referred to in Article 19-2 of the Early Childhood Education Act;  3. Other information systems prescribed by Ordinance of the Ministry of Health and Welfare, including the integrated electronic civil petition window referred to in Article 12-2 (3) of the Civil Petitions Treatment Act.  (6) Except as provided in this Act, matters concerning the protection and management of information referred to in paragraphs (1) through (5) shall be governed by the Personal Information Protection Act.  [This Article Newly Inserted on Dec. 29, 2015]  [Moved from Article 33-2 <Dec. 3, 2019>] |
| Article 40 (Stockpiling Medical and Quarantine Supplies in Preparation for Infectious Diseases Spread through Bioterrorism) | (1) When there is a likelihood of a pandemic of infectious diseases spread through bioterrorisms or any other infectious disease, the Commissioner of the Korea Disease Control and Prevention Agency may determine preventive and therapeutic medical and quarantine supplies, subject to deliberation by the Committee, and stockpile them or enter an agreement in advance for their long-term purchase. <Amended on Jan. 18, 2010; Aug. 11, 2020; Dec. 15, 2020>  (2) When there is a likelihood of a pandemic of an infectious disease spread through bioterrorism and any other infectious disease, the Commissioner of the Korea Disease Control and Prevention Agency may determine preventive and therapeutic medicines and require medicine manufacturers to produce them, notwithstanding Article 31 (2) of the Pharmaceutical Affairs Act. <Amended on Jan. 18, 2010; Dec. 3, 2019; Aug. 11, 2020>  (3) The Commissioner of the Korea Disease Control and Prevention Agency shall investigate the efficacy and adverse reactions of preventive and therapeutic medicines under paragraph (2), and conduct epidemiological investigations pursuant to Article 18 if any case of adverse reactions occurs. <Amended on Jan. 18, 2010; Aug. 11, 2020> |

Special Act on Compensation for Damage Related to COVID-19 Vaccination:

22nd (2025) Newly Established Legislative Bill (to be published)

Table S5. Medical Supply Management, Stockpiling, and Procurement: SPECIAL ACT FOR PROMOTION OF THE DEVELOPMENT AND EMERGENCY SUPPLY OF MEDICAL PRODUCTS IN RESPONSE TO PUBLIC HEALTH CRISIS

| CHAPTER I GENERAL PROVISIONS  Article 1 (Purpose) The purpose of this Act is to contribute to the protection of public safety and health by prescribing matters necessary to promote the development of medical products in response to a crisis and to create a supply base for emergency use in order to quickly overcome a public health crisis. |
| --- |
| Article 4 (Relationship to Other Statutes) Except as provided in this Act with respect to crisis response medical products, the Pharmaceutical Affairs Act shall apply to drugs and quasi-drugs, the Act on the Safety of and Support for Advanced Regenerative Medicine and Advanced Biological Products and the Pharmaceutical Affairs Act to advanced biological products, the Medical Devices Act to medical devices, and the Act on In Vitro Diagnostic Medical Devices and the Medical Devices Act to in vitro diagnostic medical devices, respectively. |
| CHAPTER II COMMISSION ON SAFETY CONTROL AND SUPPLY OF MEDICAL PRODUCTS IN RESPONSE TO PUBLIC HEALTH CRISIS Article 5 (Commission on Safety Control and Supply of Medical Products in Response to Public Health Crisis) (1) A Commission on Safety Control and Supply of Medical Products in Response to a Public Health Crisis (hereinafter referred to as the "Commission") shall be established under the control of the Ministry of Food and Drug Safety to deliberate on the following matters and to provide advice regarding the inquiry of the Minister of Food and Drug Safety:  (2) The Commission shall consist of up to 30 members including one chairperson.  (3) The Vice Minister of Food and Drug Safety shall serve as the chairperson of the Commission; and its members shall be appointed or commissioned by the Minister of Food and Drug Safety, from among public officials belonging to the Senior Executive Service of the relevant central administrative agency prescribed by Presidential Decree and persons with extensive knowledge of and experience in medical products in response to a public health crisis. In such cases, the non-public official members shall constitute the majority of the members.  (4) The Commission may have sectoral subcommittees to efficiently perform its duties.  (5) The deliberation and resolution of a subcommittee shall be reported to the chairperson and be resolved on by the Commission: Provided, That the resolution of a subcommittee may substitute for the resolution of the Commission for matters deemed minor by the chairperson.  (6) Other details necessary for the composition, operation, etc. of the Commission and subcommittees shall be prescribed by Ordinance of the Prime Minister. |
| CHAPTER III DESIGNATION OF, PERMISSION FOR, AND SAFETY CONTROL OF PRELIMINARY CRISIS RESPONSE MEDICAL PRODUCTS  SECTION 1 Designation of and Permission for Preliminary Crisis Response Medical Products  Article 6 (Designation of Preliminary Crisis Response Medical Products) (1) A person intending to develop a crisis response medical product may file an application to designate such product as a preliminary crisis response medical product with the Minister of Food and Drug Safety; in such cases, the following materials shall be submitted together:  (2) Where a medical product to be designated and developed as a preliminary crisis response medical product under paragraph (1) is used for the purpose of diagnosis, treatment, etc. of any disease or injury specified in the items of subparagraph 3 of Article 2 and meets the following requirements, the Minister of Food and Drug Safety may designate the medical product as a preliminary crisis response medical product after deliberation by the Commission:  1. The medical product shall be one of the following items and is intended to be used to diagnose, treat, mitigate, cure, or prevent the relevant disease:  (a) Drugs such as antibiotics, antivirals, antifungals, or vaccines that work against pathogens that have developed resistance to existing drugs;  (b) Drugs such as antibiotics, antivirals, antifungals, or vaccines that work against new or untreated pathogens;  (c) Medical devices that have significantly improved or are expected to improve safety and effectiveness compared to existing medical devices or therapeutic technologies or that are not essentially equivalent thereto in terms of the purpose of use, operating principle, etc.;  (d) Medical products that can diagnose, treat, mitigate, cure, or prevent a disease caused by a pandemic of an infectious disease or the use of a biochemical weapon;  (e) Medical products to protect against radiation or radioactivity;  (f) Medical products similar to those under items (a) through (e) that are determined and publicly notified by the Minister of Food and Drug Safety;  2. The medical product shall be predicted to diagnose, treat, mitigate, cure, or prevent a disease or injury that is likely to cause serious harm to public health in non-clinical or clinical trials.  (3) The Minister of Food and Drug Safety shall decide whether to make a designation under paragraph (2) within 30 days from the date of receipt of the application under paragraph (1).  (4) Where any of the following cases is applicable to a preliminary crisis response medical product designated under paragraph (2), the Minister of Food and Drug Safety may revoke the designation of such medical product after deliberation by the Commission: Provided, That the designation shall be revoked in the case of subparagraph 1:  1. Where the designation under paragraph (2) has been obtained by fraud or other improper means;  2. Where permission by item under Articles 31 (2) through (4) and 42 (1) of the Pharmaceutical Affairs Act (including permission by item under Articles 23 (2) and (3) and 27 (1) of the Act on the Safety of and Support for Advanced Regenerative Medicine and Advanced Biological Products) has been granted for drugs or quasi-drugs (hereinafter referred to as "drugs, etc.") that have improvement effects equal to or higher than those of preliminary crisis response drugs or quasi-drugs;  3. Where permission or certification for manufacturing or importing medical devices has been granted under Article 6 (2) or 15 (2) of the Medical Devices Act for medical devices that have performance and effectiveness equal to or higher than those of preliminary crisis response medical devices (including medical devices for which permission or certification for manufacturing or importing in vitro diagnostic medical devices is granted under Article 5 (3) or 11 (2) of the Act on In Vitro Diagnostic Medical Devices).  (5) To revoke the designation of a preliminary crisis response medical product under paragraph (4), a hearing shall be held.  (6) Details necessary for the procedures, methods, etc. of the designation under paragraphs (1) and (2) shall be prescribed by Ordinance of the Prime Minister. |
| Article 7 (Prioritized Review) (1) The Minister of Food and Drug Safety shall prioritize the review of a preliminary crisis response medical product over the review of other medical products, except for a good cause, in any of the following cases:  1. Where permission for manufacturing and distributing each item, permission for importing each item, manufacturing permission, import permission, manufacturing certification, or import certification (hereinafter referred to as "permission by item") has been applied (including an application for change) for the preliminary crisis response medical product;  2. Where an application for approval of a clinical trial plan (including an application for change) has been filed for the preliminary crisis response medical product.  (2) The deadline for the prioritized review under paragraph (1) and other necessary matters shall be prescribed by Ordinance of the Prime Minister. |
| Article 8 (On-Call Concomitant Review) (1) Where a person who has obtained the designation of a preliminary crisis response medical product intends to obtain permission by item for that medical product, the person may pre-submit, before filing an application for permission by item, all or part of the documentation required for such application, which is completed at each phase of the development process, to the Minister of Food and Drug Safety at each such phase and may file an application to conduct a review whenever necessary (hereinafter referred to as "on-call concomitant review") with the Minister of Food and Drug Safety.  (2) Where deeming that the application under paragraph (1) is suitable for an on-call concomitant review, the Minister of Food and Drug Safety may approve the application under paragraph (1) after consulting with the applicant regarding the type and scope of documentation to be submitted for the on-call concomitant review, the submission schedule, and the timing of notification of the review results. The same shall apply when intending to change the approved details, such as the type and scope of the documentation submitted.  (3) When an on-call concomitant review is completed for each phase, the Minister of Food and Drug Safety shall notify the applicant of the results of each review of the documentation pre-submitted, in accordance with the timing of notification of the review results agreed upon under paragraph (2); and if the results of the review are deemed sufficient to file an application for permission by item, the Minister shall notify the applicant of such fact.  (4) Where an application for permission by item is filed for a preliminary crisis response medical product for which an on-call concomitant review is completed, the Minister of Food and Drug Safety shall reflect the results of the review notified under paragraph (3) in the decision whether to grant permission by item.  (5) Details necessary for an application for review under paragraph (1), approval or approval for change under paragraph (2), notification of review results under paragraph (3), etc. shall be prescribed by Ordinance of the Prime Minister. |
| Article 9 (Concurrent Review) The Minister of Food and Drug Safety may conduct a concurrent review for permission by item in any of the following cases:  1. Where a preliminary crisis response drug and a preliminary crisis response medical device are assembled or combined together;  2. Where applications are concurrently filed for permission by item for a preliminary crisis response drug and for permission by item for a medical device for diagnosing an indication for the preliminary crisis response drug. |
| Article 10 (Support for Clinical Trials) (1) Where a person who has obtained the designation of a preliminary crisis response medical product intends to conduct clinical trials (including therapeutic exploratory trials and therapeutic confirmatory trials) or non-clinical trials (hereinafter referred to as "clinical trials, etc."; hereafter in this Article, the same shall apply) on such medical product, the Minister of Food and Drug Safety may provide support for preparation of plans for clinical trials, etc., recruitment of subjects, training of personnel, implementation of international collaborative trials, etc.  (2) To efficiently and promptly conduct clinical trials, etc. for preliminary crisis response medical products and to improve the quality of related information, the Minister of Food and Drug Safety may collect and manage necessary information on the results of clinical trials, etc. in electronic form and may provide support, including providing such information, to persons who intend to conduct clinical trials on preliminary crisis response medical products.  (3) Details necessary for the support for the preparation of plans for clinical trials, etc., recruitment of subjects, training of personnel, implementation of international collaborative trials, etc. under paragraph (1) and the procedures and methods, etc. for collecting and managing information on clinical trials, etc. under paragraph (2) shall be prescribed by Ordinance of the Prime Minister. |
| Article 11 (Conditional Permission by Item) (1) For preliminary crisis response drugs that have completed a therapeutic exploratory trial similar in form and purpose to a therapeutic confirmatory trial in terms of the safety and effectiveness of the drug and the nature of the target disease, the Minister of Food and Drug Safety may grant permission by item on the condition that the confirmatory trial data, etc. of the drugs be submitted within a specified period; in such cases, the following data shall be submitted:  1. Clinical trial data proving that the drugs have clinical effectiveness from a pharmacoepidemiologic, pharmacotherapeutic, or pathophysiologic perspective, etc. or those proving effectiveness with surrogate endpoints that can reasonably predict the clinical effectiveness of the drugs;  2. Other materials prescribed by Ordinance of the Prime Minister with regard to the safety and effectiveness of the drugs.  (2) For preliminary crisis response medical devices for which it is impractical to obtain the number of subjects for a clinical trial for the indication, the Minister of Food and Drug Safety may grant permission by item on the condition that clinical trial data that can confirm the safety and effectiveness of the medical devices be submitted within a specified period; in such cases, the following data shall be submitted:  1. Clinical trial data ascertaining that the medical devices have clinical effectiveness, even if the number of subjects is less than that for a statistically valid trial;  2. Other materials prescribed by Ordinance of the Prime Minister with regard to the safety and effectiveness of the medical devices.  (3) For preliminary crisis response medical products for which it is deemed clear that clinical trials to study their effectiveness against nuclear, biological weapons, etc. cannot be conducted, the Minister of Food and Drug Safety may grant permission by item on the condition that clinical trial data that can confirm the safety and effectiveness of the medical products be submitted within a specified period, if the following data are submitted:  1. Drugs:  (a) Explanation that the mechanism of action of the relevant drug, including how the drug reduces toxicity, is reasonable in light of pathophysiology, etc.;  (b) Evidence that the results from non-clinical trials on animals are sufficient to predict response in humans;  (c) A clear explanation that the endpoints used in non-clinical trials on animals are related to a benefit, such as increasing survivability of humans;  (d) Evidence demonstrating that pharmacokinetic and pharmacodynamic data in humans or animals are the appropriate dose for use in humans for the relevant drug for which an application has been filed;  (e) Other materials prescribed by Ordinance of the Prime Minister with regard to proving, etc. the safety and effectiveness of the relevant drug;  2. Quasi-drugs:  (a) Explanation that the mechanism of action of the relevant quasi-drug is reasonable in light of the safety and effectiveness in the treatment, mitigation, cure, or prevention of the disease in question;  (b) Evidence that the results from non-clinical trials are sufficient to predict safety and efficacy in humans;  (c) Other materials prescribed by Ordinance of the Prime Minister with regard to proving, etc. the safety and effectiveness of the relevant quasi-drug;  3. Medical devices:  (a) Explanation that the relevant medical device's principle of action is reasonable in light of the safety and effectiveness in the diagnosis, treatment, mitigation, cure, or prevention of the disease in question;  (b) Evidence that the results from non-clinical trials on animals are sufficient to predict safety and effectiveness in humans;  (c) Other materials prescribed by Ordinance of the Prime Minister with regard to proving, etc. the safety and effectiveness of the relevant medical device.  (4) A person who has received permission by item for a drug under paragraphs (1) and (3) shall be deemed a person who has received permission by item under Articles 31 (2) through (3) and 42 (1) of the Pharmaceutical Affairs Act (including permission by item under Articles 23 (2) and (3) and 27 (1) of the Act on the Safety of and Support for Advanced Regenerative Medicine and Advanced Biological Products); a person who has received permission by item for a quasi-drug under paragraph (3) shall be deemed a person who has received permission by item under Articles 31 (4) and 42 (1) of the Pharmaceutical Affairs Act; and a person who has received manufacturing or import permission or certification for a medical device under paragraphs (2) and (3) shall be deemed a person who has received manufacturing or import permission or certification for a medical device under Article 6 (2) or 15 (2) of the Medical Devices Act (including manufacturing or import permission or certification for an in vitro diagnostic medical device under Article 5 (3) or 11 (2) of the Act on In Vitro Diagnostic Medical Devices).  (5) Where a person who has received permission by item for the relevant medical product under paragraphs (1) through (3) fails to fulfill the conditions of the permission, the Minister of Food and Drug Safety shall immediately revoke such permission by item as prescribed by Ordinance of the Prime Minister: Provided, That in cases prescribed by Ordinance of the Prime Minister, such as when it is necessary to ensure treatment opportunities for those to whom the medical product are administered or for those who use the medical product, the Minster may revoke the permission by item after requiring the person to take necessary measures.  (6) Details necessary for the application methods, processing procedures, and materials to be submitted under paragraphs (1) through (5) shall be prescribed by Ordinance of the Prime Minister. |
| Article 12 (Approval for Emergency Use) (1) In cases where deeming it necessary for responding appropriately to a public health crisis or at the request of the head of the relevant central administrative agency, the Minister of Food and Drug Safety may, after deliberation by the Commission, make any of the following dispositions (hereinafter referred to as "approval for emergency use"): Provided, That deliberation by the Commission may be omitted in urgent cases:  1. Allowing a manufacturer or importer to manufacture or import and then supply drugs, etc. for which permission by item has not been obtained or a notification by item has not been filed under Article 31 (2) or (4) or 42 (1) of the Pharmaceutical Affairs Act, or Article 23 (2) or 27 (1) of the Act on the Safety of and Support for Advanced Regenerative Medicine and Advanced Biological Products: Provided, That approval for the importation and supply of drugs, etc. shall be limited for those which have been subjected to measures equivalent to permission, etc. under Article 31 or 42 of the Pharmaceutical Affairs Act or Article 23 or 27 of the Act on the Safety of and Support for Advanced Regenerative Medicine and Advanced Biological Products in a foreign country recognized by the Minister of Food and Drug Safety as implementing drug safety control at a level equal to or higher than that of the Republic of Korea;  2. Allowing a manufacturer or importer to manufacture or import and then supply drugs, etc. for which permission by item has already been obtained or a notification by item has already been filed, specifying the use, dose, efficacy, effectiveness, and duration of use that is different from the details of the permission or notification;  3. Allowing a manufacturer or importer to manufacture or import and then supply medical devices for which manufacturing or import permission or manufacturing or import certification has not been obtained, or a manufacturing or import notification has not been filed under Article 6 (2) or 15 (2) of the Medical Devices Act or Article 5 (3) or 11 (2) of the Act on In Vitro Diagnostic Medical Devices: Provided, That approval for the importation and supply of medical devices shall be limited for those which have been subjected to measures equivalent to permission, etc. under Article 6 or 15 of the Medical Devices Act or Article 5 or 11 of the Act on In Vitro Diagnostic Medical Devices in a foreign country recognized by the Minister of Food and Drug Safety as implementing medical device safety control at a level equal to or higher than that of the Republic of Korea.  (2) To grant approval for emergency use under paragraph (1), the Minister of Food and Drug Safety may request the manufacturer or importer of the relevant medical product or the head of the relevant central administrative agency to submit necessary materials.  (3) Where deeming that approval for emergency use of a medical product in a specific field is necessary to effectively overcome a public health crisis, the Minister of Food and Drug Safety may receive an application for approval for emergency use from the manufacturer or importer of a medical product by publicly announcing the following; in such cases, the manufacturer or importer of the medical product for which an application for emergency use is filed shall submit materials, etc. proving the safety and effectiveness of the medical product as prescribed by Ordinance of the Prime Minister:  1. The scope of the medical product eligible for approval for emergency use;  2. Other matters determined by the Minister of Food and Drug Safety to be necessary, such as the requirements for approval for emergency use and duration of the approval.  (4) The Minister of Food and Drug Safety may order the manufacturer or importer of a medical product approved for emergency use under paragraph (1) to take measures under Articles 13 through 15.  (5) The Minister of Food and Drug Safety may order the manufacturer or importer of a medical product approved for emergency use, the founder of a medical institution, etc. to take such necessary measures as suspension of manufacturing or importation, recall and disposal, or suspension of use, as prescribed by Presidential Decree, in any of the following cases: Provided, That in cases falling under subparagraph 2, such measures as suspension of manufacturing or importation, recall and disposal, or suspension of use shall be taken:  1. Where the public health crisis has ended;  2. Where the medical product is recognized as causing or likely to cause significant damage to public health or as having or likely to have a fatal effect on public health;  3. Where the person who manufactures or imports the medical product fails to implement measures or fulfill obligations under this Act.  (6) Where a person who has received approval for emergency use under paragraph (1) files an application for permission by item as prescribed by Ordinance of the Prime Minister, the Minister of Food and Drug Safety may grant permission by item on the condition that the evidence of the safety and effectiveness of the relevant medical product, such as the results of use of the medical product or clinical trial data be submitted within a specified period.  (7) The Minister of Food and Drug Safety may apply Articles 7 through 9 to a medical product for which an application for permission by item has been filed under paragraph (6).  (8) Articles 11 (4) and (5) and 13 through 16 shall apply mutatis mutandis to a medical product for which permission by item has been granted or a person who has received such permission by item pursuant to paragraph (6).  (9) The details necessary for the dispositions under paragraph (1), the application process and scope of materials to be submitted under paragraph (3), the measures under paragraph (5), the processing procedures under paragraph (6), and other similar matters shall be prescribed by Ordinance of the Prime Minister.  Article 12-2 (State Compensation for Damage from Side Effects of Drugs Authorized for Emergency Use) (1) Where a person who has used a drug authorized for emergency use pursuant to Article 12 (1) becomes ill, becomes disabled, or dies as a result of a side effect of such drug, the State shall pay compensation under the following subparagraphs:  1. Medical expenses:  2. Lump-sum compensation for disability;  3. Lump-sum compensation for death;  4. Funeral expenses.  (2) The Minister of Food and Drug Safety may entrust part of the business affairs related to the payment of compensation under paragraph (1) to the Korea Institute of Drug Safety and Risk Management (hereinafter referred to as the "Korea Institute of Drug Safety and Risk Management") under Article 68-3 (1) of the Pharmaceutical Affairs Act.  (3) Articles 86-3 through 86-8 of the Pharmaceutical Affairs Act shall apply mutatis mutandis to the payment of compensation under paragraph (1). In such cases, "benefits for relief of injury" shall be construed as "compensation", and "deposit them in the charges account as earnings" shall be construed as "revert to the National Treasury".  [This Article Newly Inserted on Mar. 28, 2023] |
| SECTION 2 Safety Control and Evaluation of Medical Products  Article 13 (Safe Use Measures) (1) A person who has received permission by item for a medical product under Article 11 shall conduct an investigation, etc. on the safe use measures and usage performance of the medical product and report the results to the Minister of Food and Drug Safety, as prescribed by Ordinance of the Prime Minister.  (2) Where necessary based on the results of an investigation, etc. of safe use measures and usage performance under paragraph (1), the Minister of Food and Drug Safety may order a person who has received permission by item for the relevant medical product to take additional measures to ensure safe use.  (3) Where a person who has received permission by item for a medical product fails to comply with an order to take measures under paragraph (2), the Minister of Food and Drug Safety may order the person to recall and dispose of the relevant medical product or to take other necessary measures.  (4) Where a person who has received an order under paragraph (3) fails to comply with the order, the Minister of Food and Drug Safety may authorize a relevant public official to recall and dispose of the relevant medical product or to take other necessary measures.  (5) Where ordering a person who has received permission by item for a medical product to recall and dispose of the medical product in circulation or to take other necessary measures under paragraph (3), the Minister of Food and Drug Safety may order the person to publish the fact.  (6) Details necessary for orders for recall, disposal, other measures, and publication, etc. under paragraphs (3) through (5) shall be prescribed by Ordinance of the Prime Minister. |
| Article 15 (Tracing Investigation and Registration of Details of Use) (1) The Minister of Food and Drug Safety may designate a medical product for which permission by item has been granted under Article 11 and for which it is deemed necessary to ascertain whether an adverse event occurs for a certain period of time after use as a subject of a tracing investigation and may conduct a tracing investigation for a specified period necessary for such investigation. In such cases, the Minister of Food and Drug Safety may designate an organization to conduct a tracing investigation as prescribed by Presidential Decree and entrust all or part of the tasks.  (2) A person who has received approval of a clinical trial plan or permission by item for a medical product designated as a subject of a tracing investigation under paragraph (1) or an importer of such medical product (hereinafter referred to as "person who has received permission by item for a medical product subject to a tracing investigation, etc."; hereafter in this Article, the same shall apply) shall formulate a tracing investigation plan as prescribed by Ordinance of the Prime Minister and report it to the Minister of Food and Drug Safety.  (3) Where a person who has received permission by item for a medical product subject to a tracing investigation, etc. learns that a serious adverse event has occurred to a person who used the medical product while conducting a tracing investigation in accordance with a tracing investigation plan, he or she shall investigate, analyze, and report it to the Minister of Food and Drug Safety.  (4) A medical doctor, dentist, or pharmacist who handles a medical product subject to a tracing investigation under paragraph (1) shall, with the consent of a person to use the product, register the personal information of the person to use it and the details of use with an institution designated by the Minister of Food and Drug Safety.  (5) A person who has received permission by item for a medical product subject to a tracing investigation, etc. shall register the details of the sale and supply of the relevant medical product with an institution designated by the Minister of Food and Drug Safety.  (6) The Minister of Food and Drug Safety may provide subsidies to cover all or part of the expenses incurred in conducting a tracing investigation under paragraphs (2) and (3) and filing for registration under paragraphs (4) and (5) within the budget.  (7) Where necessary based on the results of a tracing investigation, etc., the Minister of Food and Drug Safety may take measures necessary for public health on a person who has received permission by item for a medical product subject to a tracing investigation, etc., as prescribed by Presidential Decree, such as suspending clinical trials, suspending the manufacture, importation, or sale of the relevant medical product, or ordering its recall and disposal.  (8) Matters necessary for the designation of medical products subject to tracing investigations under paragraph (1), the scope, procedures, and methods of tracing investigations, the methods of reporting adverse events under paragraph (3), the procedures for consent and methods of registration under paragraph (4), the methods of registration under paragraph (5), etc. shall be prescribed by Presidential Decree. |
| Article 17 (Designation and Management of Crisis Response Medical Products) (1) To effectively prepare for a public health crisis, the Minister of Food and Drug Safety may designate and manage medical products for which permission by item has been granted or which have been certified or notified under the Pharmaceutical Affairs Act, the Act on the Safety of and Support for Advanced Regenerative Medicine and Advanced Biological Products, the Medical Devices Act, or the Act on In Vitro Diagnostic Medical Devices, as crisis response medical products, after deliberation by the Commission.  (2) Notwithstanding paragraph (1), any of the following medical products shall be deemed to be designated as a crisis response medical product under paragraph (1):  1. A medical product that has been designated as a preliminary crisis response medical product under Article 6 and for which permission by item has been granted;  2. A medical product that has been approved for emergency use under Article 12 or for which permission by item has been granted after having been approved for emergency use.  (3) In cases of designation as a crisis response medical product under paragraph (1) or in cases of being deemed to be designated as a crisis response medical product under paragraph (2), the Minister of Food and Drug Safety shall publicly announce such designation without delay and shall notify a person who manufactures, imports, or sells the medical product of the designation, management plan, etc.  (4) The Minister of Food and Drug Safety may revoke the designation of a crisis response medical product after deliberation by the Commission in any of the following cases:  1. Where the public health crisis has ended or is clearly expected to end;  2. Where it is deemed unnecessary to separately manage the medical product in case of a public health crisis due to changes in supply and demand conditions, etc.  (5) Details necessary for the methods and procedures for designation of crisis response medical products under paragraph (1), the content and methods of public announcement and notification under paragraph (3), and the revocation of designation under paragraph (4) shall be prescribed by Ordinance of the Prime Minister. |
| Article 18 (Emergency Production or Import Orders) (1) Where deeming it necessary for responding effectively to a public health crisis, or at the request of the head of the relevant central administrative agency, the Minister of Food and Drug Safety may, after deliberation by the Commission, issue an order to produce or import crisis response medical products designated under Article 17 (hereinafter referred to as "emergency production or import order") to any of the following persons (hereinafter referred to as "producers, etc."):  1. A manufacturer of drugs, etc. under Article 31 (1) or (4) of the Pharmaceutical Affairs Act or an importer of drugs, etc. under Article 42 (1) of that Act;  2. A manufacturer of advanced biological products under Article 23 (1) of the Act on the Safety of and Support for Advanced Regenerative Medicine and Advanced Biological Products or an importer of advanced biological products under Article 27 (1) of that Act;  3. A manufacturer of medical devices under Article 6 (1) of the Medical Devices Act or an importer of medical devices under Article 15 (1) of that Act;  4. A manufacturer of in vitro diagnostic medical devices under Article 5 (1) of the Act on In Vitro Diagnostic Medical Devices or an importer of in vitro diagnostic medical devices under Article 11 (1) of that Act.  (2) Producers, etc. who receive an order under paragraph (1) shall comply with such order in the absence of good cause.  (3) Where consultation, etc. among countries is necessary due to the shortage of raw materials used for the emergency production of medical products or the shortage of imported goods referred to in paragraph (1), the Minister of Food and Drug Safety may request the heads of the relevant central administrative agencies to take necessary measures pursuant to the relevant statutes and regulations. In such cases, the heads of the relevant central administrative agencies shall cooperate with such request in the absence of good cause.  (4) Where deeming it necessary for fulfilling the order under paragraph (1), the Minister of Food and Drug Safety may provide financial and administrative support to producers, etc.  (5) Where the reason for continuing the order under paragraph (1) has ceased to exist or is clearly expected to cease to exist, the Minister of Food and Drug Safety shall revoke the order without delay.  (6) Details necessary for the procedures and methods for the emergency production or import orders under paragraphs (1) through (5) shall be prescribed by Presidential Decree. |
| Article 19 (Distribution Improvement Measures) (1) In cases where it is deemed that the supply of crisis response medical products designated due to a public health crisis is significantly disrupted or at the request of the head of the relevant central administrative agency, the Minister of Food and Drug Safety may, after deliberation by the Commission, take distribution improvement measures as necessary on the medical products subject to the measures and on their sales outlets, sales procedures, sales volume, sales conditions, etc.; in such cases, the following persons shall comply therewith in the absence of good cause:  1. A pharmacy founder under Article 20 (2) of the Pharmaceutical Affairs Act;  2. A drug wholesaler under Article 45 (1) of the Pharmaceutical Affairs Act;  3. A distributor of medical devices under Article 17 (1) of the Medical Devices Act;  4. The founder of a medical institution under Article 33 of the Medical Service Act;  5. A person equivalent to those under subparagraphs 1 through 4, as determined by the Minister of Food and Drug Safety.  (2) Where the measures under paragraph (1) are related to the price stability policy, the Minister of Food and Drug Safety shall consult with the Minister of Economy and Finance in advance.  (3) Where necessary for the distribution improvement measures for medical products under paragraph (1), the Minister of Food and Drug Safety may request cooperation from the head of the relevant central administrative agency. In such cases, the head of the relevant central administrative agency shall comply therewith unless there are special reasons.  (4) Where the reason for continuing the measures under paragraph (1) has ceased to exist or is clearly expected to cease to exist, the Minister of Food and Drug Safety shall revoke the measure without delay.  (5) Details necessary for the sales procedures, the standards for sales conditions, etc., the scope of improvement measures, the revocation of the measures, etc. under paragraphs (1) through (4) shall be prescribed by Presidential Decree. |
| Article 20 (Special Cases concerning Labeling and Inspection of Crisis Response Medical Products) (1) Where deeming it necessary for supplying crisis response medical products urgently in a public health crisis, the Minister of Food and Drug Safety may exempt the following crisis response medical products from the application of all or part of the provisions specified in each relevant subparagraph, as determined by the Minister of Food and Drug Safety: <Amended on Jun. 13, 2023>  1. Drugs (excluding products under subparagraph 2) or quasi-drugs: Article 38 of the Pharmaceutical Affairs Act (limited to cases applied mutatis mutandis under Article 42 (5) of that Act) and Articles 56 through 60 of that Act;  2. Advanced biological products: Article 26 of the Act on the Safety of and Support for Advanced Regenerative Medicine and Advanced Biological Products (limited to cases applied mutatis mutandis under Article 27 (5) of that Act) and Article 31 of that Act, and Articles 57 through 60 of the Pharmaceutical Affairs Act;  3. Medical devices (excluding products under subparagraph 4): Article 13 of the Medical Devices Act (limited to cases applied mutatis mutandis under Article 15 (6) of that Act) and Articles 20 through 23, 23-2, and 24 of that Act;  4. In vitro diagnostic medical devices: Articles 13 through 15 of the Act on In Vitro Diagnostic Medical Devices, Article 13 of the Medical Devices Act (limited to cases applied mutatis mutandis under Article 15 (6) of that Act), and Articles 23 and 24 of the Medical Devices Act.  (2) Where the Minister of Food and Drug Safety decides not to apply the specified provisions on the labeling of crisis response medical products as prescribed in paragraph (1), key information such as the product name, manufacturing number, and the use-by date of the crisis response medical products shall be indicated on the containers and packaging, and other necessary information shall be provided electronically, as determined by the Minister of Food and Drug Safety. In such cases, such indication may be provided in a foreign language on the containers or packaging.  (3) Where the Minister of Food and Drug Safety decides not to apply the provisions on import management for the importer of crisis response medical products as prescribed in paragraph (1), the relevant importer shall be required to ensure the quality of the crisis response medical products by ascertaining the quality inspection results of overseas manufacturing places, etc. as determined by the Minister of Food and Drug Safety. |
| Article 20 (Special Cases concerning Labeling and Inspection of Crisis Response Medical Products) (1) Where deeming it necessary for supplying crisis response medical products urgently in a public health crisis, the Minister of Food and Drug Safety may exempt the following crisis response medical products from the application of all or part of the provisions specified in each relevant subparagraph, as determined by the Minister of Food and Drug Safety: <Amended on Jun. 13, 2023>  1. Drugs (excluding products under subparagraph 2) or quasi-drugs: Article 38 of the Pharmaceutical Affairs Act (limited to cases applied mutatis mutandis under Article 42 (5) of that Act) and Articles 56 through 60 of that Act;  2. Advanced biological products: Article 26 of the Act on the Safety of and Support for Advanced Regenerative Medicine and Advanced Biological Products (limited to cases applied mutatis mutandis under Article 27 (5) of that Act) and Article 31 of that Act, and Articles 57 through 60 of the Pharmaceutical Affairs Act;  3. Medical devices (excluding products under subparagraph 4): Article 13 of the Medical Devices Act (limited to cases applied mutatis mutandis under Article 15 (6) of that Act) and Articles 20 through 23, 23-2, and 24 of that Act;  4. In vitro diagnostic medical devices: Articles 13 through 15 of the Act on In Vitro Diagnostic Medical Devices, Article 13 of the Medical Devices Act (limited to cases applied mutatis mutandis under Article 15 (6) of that Act), and Articles 23 and 24 of the Medical Devices Act.  (2) Where the Minister of Food and Drug Safety decides not to apply the specified provisions on the labeling of crisis response medical products as prescribed in paragraph (1), key information such as the product name, manufacturing number, and the use-by date of the crisis response medical products shall be indicated on the containers and packaging, and other necessary information shall be provided electronically, as determined by the Minister of Food and Drug Safety. In such cases, such indication may be provided in a foreign language on the containers or packaging.  (3) Where the Minister of Food and Drug Safety decides not to apply the provisions on import management for the importer of crisis response medical products as prescribed in paragraph (1), the relevant importer shall be required to ensure the quality of the crisis response medical products by ascertaining the quality inspection results of overseas manufacturing places, etc. as determined by the Minister of Food and Drug Safety. |
| CHAPTER VI SUPPLEMENTARY PROVISIONS  Article 24 (Support for Research and Development) (1) The State may provide support for the research and development of crisis response medical products, such as assisting persons researching and developing crisis response medical products in conducting non-clinical or clinical trials or providing necessary research materials.  (2) The procedures and methods for research and development support under paragraph (1) and other necessary matters shall be prescribed by Ordinance of the Prime Minister. |
| Article 26 (Free Provision of Crisis Response Medical Products) (1) A person who has received permission by item for a crisis response medical product may provide it free of charge to a person who needs it.  (2) A person who intends to provide free medical products under paragraph (1) shall obtain approval of the Minister of Food and Drug Safety with a free provision plan, etc. attached, as prescribed by Ordinance of the Prime Minister.  (3) Where having granted approval under paragraph (2), the Minister of Food and Drug Safety may request cooperation on supply-related matters from the Korea Center for Rare and Essential Drugs under Article 91 (1) of the Pharmaceutical Affairs Act or the Korea Medical Device Safety Information Center. In such cases, the relevant institution shall comply therewith except in exceptional circumstances.  (4) A person who has completed the free provision of medical products under paragraph (1) shall report the results of such free provision and other matters prescribed by Ordinance of the Prime Minister to the Minister of Food and Drug Safety.  (5) Details necessary for the request for cooperation under paragraph (3), the procedures for reporting under paragraph (4), etc. shall be prescribed by Ordinance of the Prime Minister. |
| Article 28 (Fees) Any of the following persons shall pay fees prescribed by Ordinance of the Prime Minister:  1. A person who intends to obtain approval for an on-call concomitant review or approval for change under Article 8 (2);  2. A person who intends to obtain permission by item for a medical product under Article 11 or 12 (6). |
| Article 29 (Extension of Effective Period of National Stockpile of Crisis Response Medical Products) (1) Where deeming it necessary to extend the effective period of crisis response medical products stockpiled under statutes, the head of the relevant central administrative agency may request an extension of the effective period from the Minister of Food and Drug Safety.  (2) Matters necessary for the types and targets of crisis response medical products whose effective period can be requested to be extended under paragraph (1), the procedures for requesting the extension of the effective period, and the storage conditions, methods, standards, etc. shall be prescribed by Ordinance of the Prime Minister. |
| Article 30 (Establishment of Crisis Response Medical Product Information System) (1) In order to efficiently promote distribution improvement measures under Article 19 and to manage the supply of crisis response medical products, the Minister of Food and Drug Safety may establish and operate an information system.  (2) In order to efficiently promote the establishment and operation of the information system under paragraph (1), the Minister of Food and Drug Safety may request the heads of relevant central administrative agencies and manufacturers, importers, distributors, etc. of crisis response medical products to submit information necessary for distribution management, such as production, shipment, and sales volumes and sales history. In such cases, a person receiving such request shall comply therewith in the absence of good cause.  (3) The Minister of Food and Drug Safety may utilize the information system under paragraph (1) by electronically linking it with the following information systems; in such cases, the information that can be collected through the linkage shall be limited to information necessary for distribution management, such as production, shipment, and sales volumes and sales history of crisis response medical products:  1. The information system for safe use of drugs under Article 23-3 of the Pharmaceutical Affairs Act;  2. The integrated drug information system under Article 83-5 of the Pharmaceutical Affairs Act;  3. The integrated medical device information system under Article 31-3 of the Medical Devices Act;  4. Any other information system prescribed by Presidential Decree.  (4) The Minister of Food and Drug Safety may entrust business affairs necessary for the maintenance and management of information systems under paragraph (1) to the Korea Institute of Drug Safety and Risk Management. In such cases, the Minister of Food and Drug Safety may provide subsidies to cover all or part of the expenses incurred in maintaining and managing the information systems. <Newly Inserted on Mar. 28, 2023>  (5) Matters necessary for the establishment and operation of an information system under paragraphs (1), (2), and (4), a request for provision of necessary information, entrustment, etc. shall be prescribed by Presidential Decree. <Amended on March 28, 2023> |
| Article 31 (Special Cases concerning Goods Other Than Medical Products) (1) Where deeming that special measures need to be taken on goods used in conjunction with crisis response medical products in a public health crisis, the Minister of Food and Drug Safety may take necessary measures under Articles 18, 19, and 30 on the goods after consultation with the head of the central administrative agency in charge of the goods and subsequent deliberation by the Commission.  (2) Matters necessary for the consultation with the head of the central administrative agency under paragraph (1) shall be prescribed by Presidential Decree. |

Table S6. Medical Supply Management, Stockpiling, and Procurement: INFECTIOUS DISEASE CONTROL AND PREVENTION ACT

| ARTICLE | CONTENT | |  |
| --- | --- | --- | --- |
| Article 40-2 (Distribution Standards including Priority in Supplying Medical and Quarantine Supplies for Infectious Diseases) | | The Commissioner of the Korea Disease Control and Prevention Agency may determine distribution standards, including priorities in supplying medical and quarantine supplies (limited to drugs under the Pharmaceutical Affairs Act and medical devices under the Medical Devices Act) stockpiled or produced under Article 40 (1) and (2) in preparation for a pandemic of infectious diseases spread by biological terrorism or any other infectious disease, and other necessary matters, subject to deliberation by the Committee. In such cases, the Commissioner shall endeavor to establish the distribution standards to preferentially provide the medical and quarantine supplies to any of the following regions: <Amended on Aug. 11, 2020; Dec. 15, 2020; Jun. 10, 2022>  1. An area declared a special disaster area under Article 60 of the Framework Act on the Management of Disasters and Safety due to the spread of an infectious disease;  2. An area where an infectious disease is spreading, or is likely to spread, rapidly, which is prescribed by the Commissioner of the Korea Disease Control and Prevention Agency in consideration of the current status of the sickbeds, severity of patients' diseases, etc. [This Article Newly Inserted on Mar. 18, 2014] [Title Amended on Jun. 10, 2022] | |
| Article 40-3 (Export Embargoes) | | (1) Where any Class 1 infectious disease breaks out and the public health is likely to be harmed significantly due to a sudden price increase or lack of supply of products prescribed by Ordinance of the Ministry of Health and Welfare, out of medical and quarantine supplies necessary for disease prevention, quarantine, and treatment, the Minister of Health and Welfare may prohibit the relevant products from being exported or shipped out of the Republic of Korea. <Amended on Dec. 15, 2020>  (2) Where the Minister of Health and Welfare intends to impose an embargo prescribed in paragraph (1), he or she shall have a prior consultation with the heads of the relevant central administrative agencies, and determine and publicize an embargo period in advance. [This Article Newly Inserted on Mar. 4, 2020] | |
| Article 40-6 (Special Cases concerning Purchase of Vaccines and Medicines in Development in Preparation for Infectious Diseases Spread through Bioterrorism) | | (1) Where the Commissioner of the Korea Disease Control and Prevention Agency deems it difficult to address with existing vaccines or medicines against the pandemic of infectious diseases spread through bioterrorism and other infectious diseases, he or she may enter into a contract necessary for the purchase and supply of vaccines or medicines in development subject to deliberation by the Committee, notwithstanding the Act on Contracts to Which the State Is a Party.  (2) A public official shall not be subject to disciplinary action or reprimand or otherwise held liable under the State Public Officials Act or any other related statutes or regulations for the outcome of proactive performance of duties related to a contract or performance of a contract under paragraph (1), in the absence of intent or gross negligence on his or her part.  (3) The subject of and procedures for a contract under paragraph (1) and other necessary matters shall be determined by the Commissioner of the Korea Disease Control and Prevention Agency in consultation with the Minister of Economy and Finance. [This Article Newly Inserted on Mar. 9, 2021] | |
| Article 77 (Penalty Provisions) | | Any of the following persons shall be punished by imprisonment with labor for not more than five years, or by a fine not exceeding 50 million won: <Amended on Dec. 15, 2020> | |

Table S7. Social Distancing, Protection of Vulnerable Populations and Human Rights: INFECTIOUS DISEASE CONTROL AND PREVENTION ACT

| ARTICLE | CONTENT |
| --- | --- |
| Article 49 (Preventive Measures against Infectious Diseases) | 2-2. Ordering the managers, operators, users, etc. of places or facilities with a risk of spreading an infectious disease to comply with the disease control guidelines, such as preparing a list of visitors and wearing a mask;  2-3. Ordering users of means of transport likely to transmit infectious diseases, such as buses, trains, ships, and aircraft to comply with disease control guidelines, such as wearing a mask;  2-4. Ordering the observance of disease control guidelines such as wearing a mask in a specific region during a specific period as infectious diseases are likely to spread; |
| Article 49-2 (Protection Measures for Persons Vulnerable to Infection) | (1) Where a crisis alert of the caution level or higher prescribed in Article 38 (2) of the Framework Act on the Management of Disasters and Safety is issued, in order to protect low-income groups, and children, senior citizens, and persons with disabilities who use social welfare facilities, and other persons prescribed by Ordinance of the Ministry of Health and Welfare (hereinafter referred to as "persons vulnerable to infection") from respiratory infectious diseases, the Minister of Health and Welfare, a Mayor/Do Governor, or the head of a Si/Gun/Gu may take necessary measures, such as disbursing medical and quarantine supplies (limited to quasi-drugs under the Pharmaceutical Affairs) to persons vulnerable to infection. <Amended on Dec. 15, 2020>  (2) Where a crisis alert of attention level or higher is issued under Article 38 (2) of the Framework Act on the Management of Disasters and Safety, the Commissioner of the Korea Disease Control and Prevention Agency, a Mayor/Do Governor, or the head of a Si/Gun/Gu may order to take necessary measures such as disinfection of the social welfare facilities defined in subparagraph 4 of Article 2 of the Social Welfare Services Act that are used by persons vulnerable to infection. <Newly Inserted on Mar. 9, 2021>  (2) Matters necessary for the types of infectious diseases, the scope of persons vulnerable to infection, the procedures for provision, and other relevant matters under paragraph (1) shall be prescribed by Ordinance of the Ministry of Health and Welfare. <Amended on Mar. 9, 2021>  [This Article Newly Inserted on Mar. 4, 2020] |
| Article 34 (Formulation and Implementation of Crisis Control Measures against Infectious Diseases) | 5-2. Response measures for persons vulnerable to infection and social welfare facilities by type, based on the status of the outbreak and transmission of infectious diseases; |

Table S8. Infectious Disease R&D: INFECTIOUS DISEASE CONTROL AND PREVENTION ACT

| ARTICLE | CONTENT |
| --- | --- |
| Article 8-6 (Support for Research and Development of Infectious Diseases) | (1) For the survey and research of infectious diseases, the Commissioner of the Korea Disease Control and Prevention Agency may formulate plans for research and development regarding infectious diseases and implement projects of research and development of medicines, vaccine, and others. In such cases, the Commissioner of the Korea Disease Control and Prevention Agency may pay contributions to the institutions or organizations conducting research and development projects, for the expenses incurred in such research, within the budget.  (2) For the survey and research under paragraph (1), the Commissioner of the Korea Disease Control and Prevention Agency may designate a specialized institution or revoke the designation thereof pursuant to subparagraph 4 of Article 2 of the National Research and Development Innovation Act, as prescribed by Ordinance of the Ministry of Health and Welfare.  (3) The Health and Medical Service Technology Promotion Act shall apply mutatis mutandis to matters necessary for the payment, use, and management of contributions under paragraph (1) and the designation, operation, etc. of specialized institutions under paragraph (2).  (4) The Commissioner of the Korea Disease Control and Prevention Agency may conduct testing and analysis for the development of medicines and vaccines for infectious diseases at the request of the research institutes, universities, enterprises, etc. related to the development of medicines and vaccines for infectious diseases, as prescribed by Ordinance of the Ministry of Health and Welfare.  (5) Any person who requests the testing and analysis pursuant to paragraph (4) shall pay fees, as prescribed by Ordinance of the Ministry of Health and Welfare. [This Article Wholly Amended on May 19, 2023] |
| Article 76-4 (Analysis of and Research on Information on Infectious Diseases) | (1) If necessary for preventing and controlling infectious diseases and blocking the spread of infection, the Commissioner of the Korea Disease Control and Prevention Agency may analyze the following information or use such information for infectious disease-related research:  1. Information collected through reporting under Articles 11 (5) and 13; 2. Information from epidemiological investigations under Article 18; 3. Information on vaccination records under Article 28; 4. Information from epidemiological investigations on vaccination under Article 29; 5. Information provided pursuant to Article 76-2 (1) and (2); 6. Other information necessary for preventing and controlling infectious diseases and blocking the spread of infection, which is determined by the Commissioner of the Korea Disease Control and Prevention Agency.  (2) Where the Commissioner of the Korea Disease Control and Prevention Agency uses personal information pursuant to paragraph (1), he/she shall pseudonymize such information as defined in subparagraph 1-2 of Article 2 of the Personal Information Protection Act (hereafter in this Article, referred to as "pseudonymization"): Provided, That this shall not apply to any of the following cases:  1. Where the assignment of hospital beds and other urgent measures are required, allowing no time for pseudonymization; 2. Where the use of pseudonymized personal information makes it difficult to respond to adverse reactions to vaccinations, control sequelae of infectious diseases, support persons vulnerable to infection, and perform other uninterrupted work. (3) Where any personal information is used pursuant to paragraph (1), matters necessary for the legal basis, purposes, scope, etc. thereof shall be published in the Official Gazette or on the website pursuant to Article 18 (4) of the Personal Information Protection Act: Provided, That this shall not apply where pseudonymized information is used pursuant to the main clause, with the exception of the subparagraphs, of paragraph (2).  [This Article Newly Inserted on Mar. 28, 2023] [Moved from Article 76-3; previous Article 76-4 moved to Article 76-5 <Jan. 23, 2024>] Article 76-5 (Provisions Applicable Mutatis Mutandis) Article 42 (6) shall apply mutatis mutandis |
